# Matrix Architecture and Mechanics Regulate Myofibril Organization, Costamere Assembly, and Contractility in Engineered Myocardial Microtissues

**DOI:** 10.1002/advs.202309740

**Published:** 2024-11-18

**Authors:** Samuel J. DePalma, Javiera Jilberto, Austin E. Stis, Darcy D. Huang, Jason Lo, Christopher D. Davidson, Aamilah Chowdhury, Robert N. Kent, Maggie E. Jewett, Hiba Kobeissi, Christopher S. Chen, Emma Lejeune, Adam S. Helms, David A. Nordsletten, Brendon M. Baker

**Affiliations:** ^1^ Department of Biomedical Engineering University of Michigan Ann Arbor MI 48109 USA; ^2^ Department of Mechanical Engineering Boston University Boston MA 02215 USA; ^3^ Department of Biomedical Engineering Boston University Boston MA 02215 USA; ^4^ Wyss Institute for Biologically Inspired Engineering Harvard University Boston MA 02115 USA; ^5^ Division of Cardiovascular Medicine University of Michigan Ann Arbor MI 48109 USA; ^6^ Department of Cardiac Surgery University of Michigan Ann Arbor MI 48109 USA; ^7^ Department of Biomedical Engineering School of Imaging Sciences and Biomedical Engineering King's College London King's Health Partners London SE1 7EH UK; ^8^ Department of Chemical Engineering University of Michigan Ann Arbor MI 48109 USA

**Keywords:** biomaterials, cardiac tissue engineering, cardiomyocytes, electrospinning, extracellular matrix, induced pluripotent stem cells, mechanosensing

## Abstract

The mechanical function of the myocardium is defined by cardiomyocyte contractility and the biomechanics of the extracellular matrix (ECM). Understanding this relationship remains an important unmet challenge due to limitations in existing approaches for engineering myocardial tissue. Here, they established arrays of cardiac microtissues with tunable mechanics and architecture by integrating ECM‐mimetic synthetic, fiber matrices, and induced pluripotent stem cell‐derived cardiomyocytes (iPSC‐CMs), enabling real‐time contractility readouts, in‐depth structural assessment, and tissue‐specific computational modeling. They found that the stiffness and alignment of matrix fibers distinctly affect the structural development and contractile function of pure iPSC‐CM tissues. Further examination into the impact of fibrous matrix stiffness enabled by computational models and quantitative immunofluorescence implicates cell‐ECM interactions in myofibril assembly, myofibril maturation, and notably costamere assembly, which correlates with improved contractile function of tissues. These results highlight how iPSC‐CM tissue models with controllable architecture and mechanics can elucidate mechanisms of tissue maturation and disease.

## Graphical abstract

Arrays of cardiac microtissues with tunable mechanics and architecture are created by integrating extracellular matrix‐mimetic, synthetic fiber matrices, and induced pluripotent stem cell‐derived cardiomyocytes (iPSC‐CMs). Real‐time contractility readouts, in‐depth structural assessment, and tissue‐specific computational modeling reveal that stiffness and alignment of matrix fibers distinctly affect tissue structural development and contractile function, thus informing the design of translatable regenerative cardiac therapies.

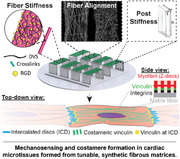

## 1 Introduction

Heart disease remains the leading cause of death worldwide.^[^ ^1^ ^]^ Despite many recent advances, existing therapies for treating heart disease fail to restore normal function of the heart following chronic or acute injury, due in part to the limited regenerative potential of the myocardium.^[^ ^2^, ^3^ ^]^ Thus, there is a critical need for regenerative or tissue‐replacement therapies that restore normal cardiac architecture and mechanical function. In recent years, advances in induced pluripotent stem cell (iPSC) technologies have made the creation of engineered heart tissues (EHTs) feasible for use as regenerative therapies, in vitro models to study cardiac regeneration, or screening platforms to test the effectiveness and/or toxicity of new therapeutics.^[^ ^4^, ^5^, ^6^ ^]^

Among the many techniques explored to generate mature iPSC‐derived cardiomyocyte (iPSC‐CM) tissues, significant efforts have focused on developing scaffolds that recapitulate physiologic tissue organization to improve overall tissue function and potentially maturity.^[^ ^7^, ^8^, ^9^ ^]^ The mechanical function of the myocardium is dictated by contractile CMs and the surrounding fibrous extracellular matrix (ECM) that organizes and supports CMs.^[^ ^10^, ^11^, ^12^ ^]^ Individual layers of muscle tissue throughout the myocardium are highly anisotropic, driving coordinated uniaxial contractions.^[^ ^13^ ^]^ These muscle fibers and their accompanying ECM twist transmurally, thus generating the torsional contractile behavior critical to the proper systolic function of the ventricle.^[^ ^14^ ^]^ As such, scaffolds that recapitulate biochemical and mechanical features of native cardiac ECM and direct cellular orientation hold promise for improving the function and maturation of cardiac tissue constructs.^[^ ^15^, ^16^, ^17^, ^18^, ^19^, ^20^ ^]^ Scaffolding is often integrated into EHTs by combining naturally derived biomaterials such as purified collagen or fibrin with iPSC‐CMs.^[^ ^8^, ^20^, ^21^, ^22^, ^23^, ^24^ ^]^ However, these materials provide limited control over mechanical properties, which is critical to investigating how iPSC‐CMs sense and respond to matrix stiffness. Moreover, the addition of admixed stromal cells is often required to drive proper tissue assembly in these systems, precluding the direct study of CM behavior. Prior studies have explored the use of polymeric hydrogels or elastomeric materials that provide improved mechanical control or alternatively, electrospun polymeric scaffolds possessing fibrous topography that better recapitulates the native ECM; however, these materials lack fibrous topography or sufficient tunability, respectively, both of which we view as potentially critical mechanobiological inputs that may influence cardiac tissue assembly or maturation.^[^ ^16^, ^17^, ^19^, ^25^, ^26^, ^27^ ^]^ Additionally, 2D micropatterned iPSC‐CM tissues often are not stable for more than 8–10 days due to inadequate adhesion formation, thus limiting their use in studying long‐term processes such as during maturation or disease.^[^ ^18^ ^]^ Thus, stable EHTs formed from iPSC‐CMs and highly tunable, fibrous matrices providing orthogonal control over architecture and mechanics could provide new and important insights into how iPSC‐CMs respond to physical microenvironment inputs. Furthermore, a deeper understanding of how CMs interact with their physical microenvironment through specialized cell‐ECM adhesions, for example, could in turn establish key design attributes of scaffolds that support stem cell‐derived cardiac tissue formation and maturation.

The interactions between cardiomyocytes and their surrounding native ECM or a biomaterial scaffold are regulated by cellular mechanosensing and ultimately the transduction of mechanical forces into cell signaling cascades. CM mechanosensing has been shown to be critical in cardiac development, disease progression, and the assembly of in vitro engineered heart tissues, highlighting the necessity for biologically informed design of scaffolds used to engineer mature iPSC‐CM tissues.^[^ ^17^, ^28^, ^29^, ^30^, ^31^, ^32^ ^]^ As CMs have extremely dynamic mechanical functions, they use multiple mechanosensing mechanisms to sense and respond to changes in their mechanical environment.^[^ ^28^ ^]^ Forces generated by the myofibrils in CMs are transmitted to neighboring cells through specialized cell‐cell adhesions termed intercalated discs (ICDs) and to the surrounding matrix through cell‐matrix linkages.^[^ ^29^, ^33^, ^34^, ^35^ ^]^ Composed of adherens junctions and desmosomes, ICDs enable mechanical and electrical coupling of neighboring CMs and help to regulate the mechanical function of the heart.^[^ ^34^ ^]^ For the myocardium to contract uniformly, CMs not only must connect to one another but also to the surrounding matrix via integrins and associated complexes of cell‐matrix adhesion proteins. In striated muscle cells such as cardiomyocytes, cell‐matrix adhesions can be grouped into two categories: peripheral FAs, which link the myofibrillar cytoskeleton to the ECM and are generally found at the edges of cultured CMs, and costameres, specialized adhesion complexes that directly link the myofibril to the surrounding or underlying matrix through connections at the z‐disc.^[^ ^29^ ^]^ During myofibril formation, peripheral FAs become load‐bearing protocostameres, followed by α‐actinin accumulation corresponding to the assembly of myofibrils.^[^ ^32^, ^36^, ^37^ ^]^ As myofibrils begin to assemble and mature, costameres replace protocostameres and tether z‐discs to the matrix.^[^ ^37^, ^38^ ^]^ Since initially described as vinculin‐containing structures linking z‐discs to the surrounding ECM, numerous other proteins commonly associated with FA complexes such as talin, paxillin, and FAK have been identified at costameres.^[^ ^32^, ^38^, ^39^, ^40^, ^41^ ^]^ Many of these proteins have also been implicated in fundamental cell processes such as myofibril assembly and tissue mechanosensing. However, how ECM and tissue mechanics impact the formation of these structures and their impact on tissue maturation and disease progression is not well understood, in due to the limited tunability and long‐term stability of existing in vitro models.

Genetic variants that impact the function of mechanosensing proteins can render cardiomyocytes abnormally susceptible to mechanical stresses, leading to ventricular hypertrophy or dilation and ultimately heart failure.^[^ ^42^, ^43^, ^44^ ^]^ In particular, the mechanosensitive protein vinculin is known to play a critical role at both costameres and intercalated discs during cardiac development and disease.^[^ ^30^, ^36^, ^45^, ^46^, ^47^, ^48^ ^]^ Genetic variants of vinculin contribute to cardiomyopathies^[^ ^43^ ^]^ and cardiomyocyte‐specific knock‐out of vinculin leads to early cardiac failure or dilated cardiomyopathies.^[^ ^47^, ^48^ ^]^ Conversely, overexpression of vinculin in drosophila hearts promotes myocardial remodeling and improved cardiac function in aging hearts, resulting in extended organismal lifespan.^[^ ^31^ ^]^ Moreover, the mechanosensitivity of vinculin in CMs has been described both in vitro and in vivo, suggesting that vinculin localization to costameres and intercalated discs regulates myofibril maturation and is influenced by mechanical signals.^[^ ^26^, ^30^, ^46^, ^49^, ^50^ ^]^ Despite these advances in our knowledge of vinculin's role in CM mechanosensing, it is unknown how fibrous matrix mechanics impact vinculin's localization to cell‐cell or cell‐ECM adhesions, or how the formation and maturation of these complexes more broadly impact EHT function and maturation.

To study how specific and tissue‐relevant structural and mechanical cues individually impact engineered cardiac tissue assembly, cell‐ECM interactions, iPSC‐CM maturation, and tissue function, we established a biomaterial‐based platform for creating arrays of cardiac microtissues composed of tunable, synthetic, fibrous ECM and purified iPSC‐CMs. Through carefully controlled studies varying ECM organization and mechanics, we found that microtissues formed on soft (< 1 kPa), aligned fibrous matrices tethered between soft elastic posts demonstrate improved CM adhesion, organization, and contractile function. Tissue‐specific computational modeling revealed that altered cellular mechanical behavior, in conjunction with the passive mechanics of the tissue, drives the observed changes in tissue contractility. Associated with these effects, we found that vinculin localization to costameres during tissue formation was dependent on fibrous matrix stiffness. Moreover, robust vinculin localization to costameres was strongly associated with tissue maturation and increases in contractile function. These findings highlight the importance of highly controlled bioengineered platforms for studying CM mechanosensing and provide several key insights that inform the design of biomaterial scaffolds for engineered cardiac tissue replacement therapies.

## 2 Results

### 2.1 Development and Characterization of Mechanically Tunable Engineered Heart Tissue Platform

As the myocardial microenvironment plays a vital role in both cardiac development and disease progression, constructing EHTs with biomaterials that recapitulate relevant architecture and mechanics of the fibrous cardiac ECM is crucial for studying these processes in vitro.^[^ ^9^ ^]^ We previously developed matrices composed of synthetic dextran vinyl sulfone (DVS) polymeric fibers that possess comparable geometry to perimysial collagen fibers, which are ≈1 µm in diameter and surround CM bundles to confer tissue mechanical anisotropy and enable the mechanical function of cardiac tissue.^[^ ^10^, ^11^, ^12^, ^15^ ^]^ Using this biomaterial platform, we showed that matrix fiber alignment is critical to driving proper tissue organization and calcium handling dynamics.^[^ ^15^ ^]^ Additionally, we found that these fibrous scaffolds facilitate long‐term culture of iPSC‐CMs (>28 days) enabled by robust cell‐matrix interactions.^[^ ^15^ ^]^ However, this culture platform did not allow for tissue fractional shortening, thereby preventing the assessment of the contractile function of formed EHTs. Predicated on this previous work, here we advanced this model by generating a platform enabling fractional shortening and orthogonal mechanical tunability to explore the impact of an expanded array of architectural and mechanical cues on iPSC‐CM function.

**Figure 1:**
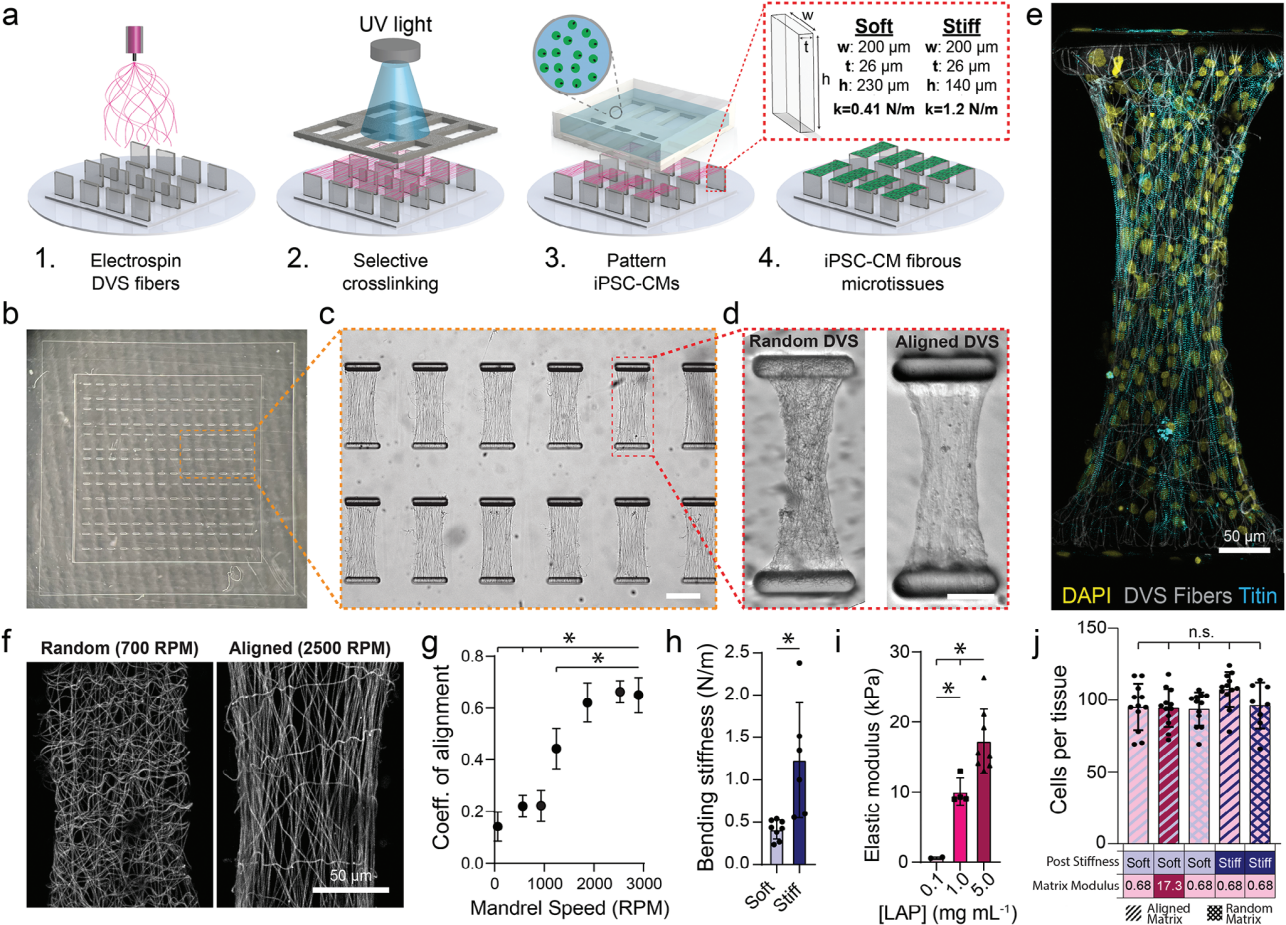
Fabrication of pure iPSC‐CM microtissues with high mechanical tunability. a) Schematic of fibroTUG fabrication and seeding. b) Full array of microfabricated PDMS posts before DVS fiber electrospinning. c) Brightfield image of photopatterned suspended matrices (scale bar: 200 µm). d) Representative brightfield images of pure populations of iPSC‐CMs seeded on random and aligned DVS matrices 7 days after seeding (scale bar: 100 µm). e) Confocal fluorescent image of fibroTUG tissue formed on 0.68 kPa, aligned matrices suspended between soft posts. f) Confocal fluorescent images of random and aligned fiber matrices functionalized with methacrylated rhodamine. g) The rotation speed of the collection mandrel during fiber electrospinning was varied to define fiber alignment (n ≥ 5 matrices). h) Post height was varied to define post bending stiffness. i) LAP photoinitiator concentration was tuned to generate matrices of physiologically relevant stiffnesses. j) Tissue seeding was unaffected by the mechanical inputs, as quantified by the number of cells that compose each tissue 7 days after seeding. All data presented as mean ± std; ^*^ *p* < 0.05.

To examine how variations in the architecture and mechanics of the cardiac microenvironment influence iPSC‐CM tissue formation, we established a new approach to generating arrays of cardiac microtissues (termed fibroTUGs or fibrous tissue µ‐gauges) composed of tunable electrospun, synthetic fiber matrices suspended between two elastomeric posts seeded with pure populations of iPSC‐CMs using a microfabrication‐based cell patterning strategy. Microfabricated PDMS post arrays consisting of 98 pairs of rectangular posts were fabricated with standard soft lithography techniques (**Figure** **1**a,b). Based on previous literature^[^ ^22^, ^51^ ^]^ and as confirmed via custom mechanical characterization methods (Figure S1a–c, Supporting Information), post heights were defined to generate soft (0.41 N m^−1^) and stiff (1.2 N m^−1^) mechanical boundary conditions (Figure 1a,h). Subsequently, electrospun DVS fibers were deposited upon post arrays affixed to a collecting mandrel rotating at various speeds to control fiber alignment, as previously described^[^ ^15^ ^]^ (Figure 1a,f,g). Next, fiber matrices spanning two posts were stabilized via photoinitiated free radical crosslinking by exposing substrates to UV light through a photopatterning mask in the presence of lithium phenyl‐2,4,6‐trimethylbenzoylphosphinate (LAP) photoinitiator. Upon hydration, uncrosslinked fibers were dissolved, resulting in fibrous matrices spanning only between pairs of posts. Stabilized fibrous matrices spanning posts could then be crosslinked further via additional exposure to UV light in the presence of LAP to define a final matrix stiffness.^[^ ^15^, ^52^ ^]^ Crosslinking parameters were identified to generate matrices with stiffnesses corresponding to developing (0.1 mg mL^−1^ LAP; 0.68 kPa), adult (1.0 mg mL^−1^ LAP; 10.1 kPa), or diseased (5.0 mg mL^−1^ LAP; 17.4 kPa) myocardium, as characterized by microindentation measurements (Figure 1i; Figure S1d–f, Supporting Information).^[^ ^17^, ^53^, ^54^ ^]^ To generate pure cardiomyocyte tissues without the requirement of admixed stromal cells, purified cultures of iPSC‐CMs (≥95% TTN+ CMs; Figure S2, Supporting Information) were seeded upon photopatterned matrices using physically registered, microfabricated seeding masks that funneled iPSC‐CMs to the suspended matrices, limiting seeded cells from settling and adhering to the glass surface below suspended fiber matrices (Figure 1a). The resulting tissues contained ≈100 iPSC‐CMs regardless of the pre‐defined mechanics of the posts or matrices (Figure 1j).

While matrix stiffness has been extensively studied using polymeric hydrogel or elastomer surfaces,^[^ ^17^, ^18^, ^26^, ^41^, ^54^ ^]^ little is known about how the stiffness of assemblies of fibers influences iPSC‐CM tissue formation and function. The discrete nature of fibrous matrices engenders distinct behavior compared to elastic, continuum‐like materials.^[^ ^55^ ^]^ Although here we provide measurements of bulk modulus to demonstrate mechanical tunability, these values cannot be directly extrapolated to continuum‐like materials. Of note, these matrix fibers are individually quite stiff (≈100′s of MPa) despite the soft bulk stiffness of the overall matrix given its high void fraction. This is in contrast to hydrogel or elastomer materials where mechanics are fairly uniform across bulk to cell length‐scales.^[^ ^52^ ^]^ The cell‐scale mechanics of these fibers, which we view as most relevant to CM mechanosensing, are therefore within the range of values reported for common natural biomaterials such as perimysial collagen fibers in the heart.^[^ ^56^, ^57^, ^58^ ^]^ Furthermore, because our fibroTUG platform is predicated upon predefined, nondegradable synthetic matrices that facilitate robust integrin engagement to guide the assembly of myocardial syncytia structurally similar to stratified muscle layers in the myocardium, we were able to generate and monitor functional myocardial tissues composed of pure iPSC‐CMs for greater than 3 weeks, highlighting the utility of this system in studying the impact of mechanical cues on cell‐ and tissue‐scale function over long‐term culture (Figures S3,S11, and S12, Supporting Information).^[^ ^7^ ^]^

To demonstrate the general utility of this mechanically tunable platform for studying microenvironmental inputs on other types of tissues beyond EHTs, we also seeded fibroTUGs with varying fiber stiffness with tendon progenitor cells (TPC) to examine whether fiber stiffness influences tenogenic differentiation (Supporting Information).^[^ ^59^ ^]^ We found that TPCs cultured on stiff fibers expressed higher levels of scleraxis, a key marker of tenogenic differentiation (Figure S4, Supporting Information). Additionally, treatment of tissues on stiff matrices with pro‐tenogenic TGF‐β3 resulted in a further increase in tenogenic differentiation, supporting the general observation that a combination of physical and soluble cues potentiate stem cell differentiation (Figure S4, Supporting Information). These results highlight the broad applicability of highly tunable microtissue platforms for understanding cellular mechanosensing in a variety of tissue contexts.

### 2.2 Mechanical Inputs Impact Tissue Mechanical Function, Organization, and Maturation

**Figure 2:**
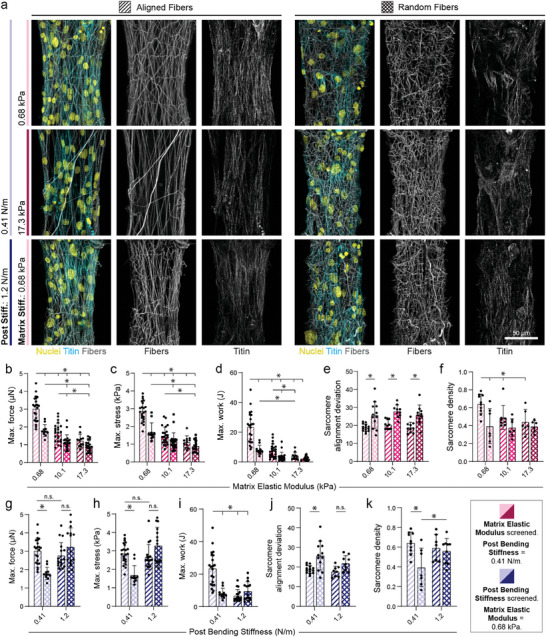
Fibrous matrix alignment and stiffness influence iPSC‐CM tissue assembly and force generation. a) Confocal fluorescent images of fibroTUG tissues of varied fiber alignment, fiber stiffness, and post stiffness seeded with iPSC‐CMs possessing a GFP‐TTN reporter. All images show a region located at the center of each tissue. Maximum contractile b) force, c) stress, and d) work quantified in tissues with constant post stiffness (0.41 N m^−1^) with varied fiber alignment and fiber stiffness (n ≥ 11 tissues). e) Sarcomere alignment deviation and f) sarcomere density quantified in tissues with constant post stiffness (0.41 N m^−1^) and varying fiber alignment and fiber stiffness (n ≥ 10 tissues). Maximum contractile g) force, h) stress, and i) work quantified in tissues with constant matrix stiffness (0.68 kPa) with varied fiber alignment and post stiffness (n ≥ 11). j) Sarcomere alignment deviation and k) sarcomere density quantified in tissues with constant matrix stiffness (0.68 kPa) with varied fiber alignment and post stiffness (n ≥ 8). All data presented as mean ± std; ^*^ *p* < 0.05.

After thorough mechanical characterization of our fibroTUG platform, we examined how altering key architectural and mechanical inputs – specifically matrix alignment, matrix stiffness, and post stiffness – affect iPSC‐CM tissue assembly, organization, and function (**Figure** **2**). Tuning each of these parameters orthogonally enables the controlled study of their respective influence on EHT formation and function, thereby providing insights into how CMs respond to (patho)physiologically relevant microenvironmental factors. For example, disorganized collagenous ECM found in fibrotic scars after myocardial infarction has been suggested to promote disease by mitigating CM contractility and inducing pathogenic signaling.^[^ ^60^, ^61^, ^62^, ^63^, ^64^ ^]^ ECM stiffness has similarly been shown to increase dramatically in fibrotic conditions, while gradually increasing throughout heart development and maturation.^[^ ^53^, ^62^, ^63^, ^64^, ^65^ ^]^ Finally, changes in post stiffness model changes in tissue afterload that occur throughout tissue development concurrent with CM maturation and are further increased in various forms of cardiac disease.^[^ ^51^, ^66^ ^]^ While previously established EHT platforms enable the study of some of these mechanical perturbations, our system allows for orthogonal tuning of all these inputs in fibrous matrices that better recapitulate perimysial collagen network. This ability opens the door to understanding how each input alone impacts tissue function and how combinations of these inputs may impact tissue signaling involved in iPSC‐CM maturation. To assess the resulting tissue function in various mechanical environments, the contractility of tissues after 7 days of culture was quantified by measuring post deflections from time‐lapse imaging of contracting tissues (Videos S1–S5, Supporting Information). Myofibril organization and density were quantified as previously described to assess the impact of physical microenvironmental cues on tissue organization.^[^ ^15^, ^67^ ^]^

Regardless of matrix conditions, all tissues contracted uniaxially, as evidenced by inward post deflections (Videos S1–S9, Supporting Information), similar to established rectangular micropatterned 2D and 3D tissues.^[^ ^17^, ^18^, ^21^, ^22^, ^26^ ^]^ Examining fibroTUGs formed on aligned fibrous matrices spanning soft (0.41 N m^−1^) posts, we noted a decrease in tissue contractile force, contractile stress, and work as a function of increasing matrix stiffness (Figure 2b–d; Video S2, Supporting Information). Exploring the effect of matrix fiber alignment (via pre‐defining aligned vs randomly oriented fibers), we noted diminished tissue contractility on soft (0.68 kPa) matrices with randomly oriented fibers as compared to matrices composed of aligned fibers of equivalent fiber stiffness (Figure 2b–d; Videos S2 and S3, Supporting Information). On stiffer matrices, differences in tissue stress arising from fiber alignment were less prominent, potentially indicating that iPSC‐CMs may be unable to deform stiffer matrices or efficiently assemble myofibrils independent of matrix fiber alignment. Additionally, contraction and relaxation velocities were highest on soft, aligned matrices (Figure S5a,b, Supporting Information). We next quantified myofibril organization within these tissues and observed a decrease in sarcomere alignment on randomly oriented fiber matrices regardless of matrix stiffness, as quantified by a higher sarcomere deviation^[^ ^15^ ^]^ (Figure 2a,e,f). Additionally, sarcomere density decreased on random matrices compared to aligned matrices across all stiffnesses tested (Figure 2a,e,f). Finally, we examined the influence of tissue boundary stiffness on resulting tissue contractility by maintaining a constant stiffness of aligned matrices at 0.68 kPa while increasing post stiffness (Figure 2g–i; Videos S4 and S5, Supporting Information). While contractile force and stress remained constant in tissues formed between both soft (0.41 N m^−1^) and stiff (1.2 N m^−1^) posts, the effective work produced by tissues contracting against stiffer boundaries was greatly reduced (Figure 2g–i). Contraction and relaxation velocity also decreased in tissues formed between stiff (1.2 N m^−1^) posts (Figure S5e,f, Supporting Information). Contraction and relaxation durations were longer on soft, aligned matrices between soft posts due to the significantly larger fractional shortening observed in these tissues (Figure S5c,d,g–l, Supporting Information). However, tissues formed on randomly oriented, soft fiber matrices tethered between stiff posts surprisingly revealed no differences in contractile force and stress as compared to aligned, soft matrices suspended between stiff posts (Figure 2g,h). This finding may be explained by a greater relative influence of increased uniaxial workload against the stiff posts, given that these tissues also exhibited enhanced myofibril assembly and alignment compared to tissues formed on random matrices under soft boundary conditions (Figure 2,j,k). This surprising result suggests that iPSC‐CMs contracting against stiffer boundary constraints can form aligned myofibrils regardless of the topographical alignment of the underlying matrix, as has been shown previously in 3D tissues.^[^ ^21^, ^68^, ^69^ ^]^ However, as these tissues demonstrated limited fractional shortening and work, this condition may more represent a diseased mechanical environment.^[^ ^51^ ^]^

**Figure 3:**
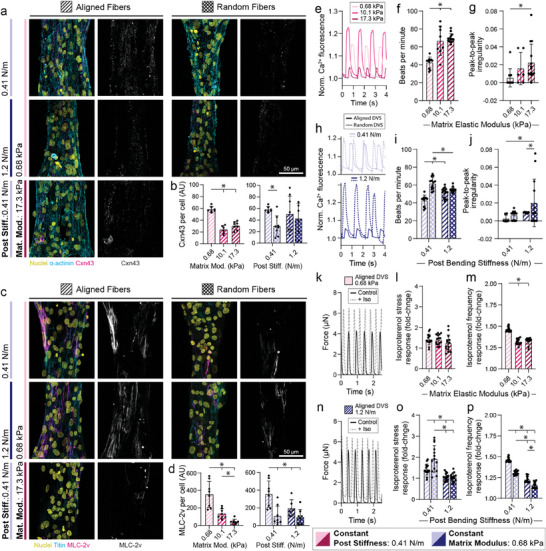
Fibrous matrix alignment, stiffness, and mechanical constraints influence iPSC‐CM tissue development. a) Confocal fluorescent images of fibroTUG tissues of varied fiber alignment, fiber stiffness, and post‐stiffness immunostained for α‐actinin and connexin‐43. All images show a region located at the center of each tissue. b) Quantification of connexin‐43 (Cxn43) expression (n ≥ 6). c) Confocal fluorescent images of fibroTUG tissues of varied fiber alignment, fiber stiffness, and post stiffness seeded with iPSC‐CMs containing a GFPtitin reporter and immunostained for MLC‐2v. Again, all images show a region located at the center of each tissue. d) Quantification of MLC‐2v expression (n ≥ 6). Calcium flux dynamics were analyzed, with representative flux traces shown in e,h), to determine f,i) contraction frequency (n ≥ 8) and g,j) peak‐to‐peak irregularity, as quantified by the standard deviation of the time interval between peaks (n ≥ 6). Contractile dynamics in response to isoproterenol treatment (10 nm) were analyzed, with representative contraction traces shown in k,n), to determine the fold change in l,o) contractile force and m,p) contractile frequency (n ≥ 14). Blue hatching within bar plots indicates where post stiffness was held constant at 0.41 N m^−1^ in (b,d,e–g,k–m) to explore the impact of matrix alignment and post stiffness on tissue development. Pink hatching within bar plots indicates matrix stiffness was held constant at 0.68 kPa in (b,d,h–j,n–p) to explore the impact of matrix alignment and fiber stiffness on tissue development. All data presented as mean ± std; ^*^ *p* < 0.05.

We next explored how microenvironmental mechanics impact iPSC‐CM EHT maturation. To assess structural maturation, we immunostained tissues formed in different mechanical environments for connexin‐43, the predominant cardiac gap junction protein, and the myosin regulatory light chain MLC‐2v, which is known to be enriched in adult ventricular CMs^[^ ^7^ ^]^ (**Figure** **3**a–d). Corroborating our measurements of fibroTUG contractility and myofibril assembly, tissues formed on soft (0.68 kPa), aligned matrices and soft (0.41 N m^−1^) posts expressed the highest levels of connexin‐43 and MLC‐2v compared to tissues formed on stiff aligned matrices, soft non‐aligned matrices, or between stiff posts (Figure 3a–d). Long‐term culture of tissues under these conditions in oxidative phosphorylation promoting media (“OxPhos” media^[^ ^18^ ^]^) yielded a progressive increase in both MLC‐2v and connexin‐43 expression until day 21 (Figure S6, Supporting Information). Additionally, cardiac troponin T expression was also most abundantly expressed in tissues formed on soft (0.68 kPa) aligned matrices and soft (0.41 N m^−1^) posts (Figure S7, Supporting Information).

To further corroborate these findings, we next assessed the calcium handling of tissues formed with the fibroTUG platform. Briefly, tissues were incubated with a calcium‐sensitive dye and imaged at high frame rates (> 65 frames/s). Quantification of calcium flux dynamics indicated tissues formed on soft (0.68 kPa) aligned matrices with soft (0.41 N m^−1^) posts contracted at the lowest frequency and at the most regular intervals (in contrast to heightened peak‐to‐peak irregularity observed on tissues formed on stiff or random matrices, suggestive of heightened arrythmogenic activity); of note, decreased contraction frequency and regularity are both considered to be characteristic of more mature iPSC‐CMs^[^ ^7^ ^]^ (Figure 3e–j; Videos S6–S8, Supporting Information). Additional analysis indicates that increased matrix alignment and post stiffness also results in changes in flux rise time, decay time, and full width half max, with soft, aligned matrices between soft posts tissues exhibiting shorter rise time and decay time than tissues formed on random matrices between stiff posts (Figure S8, Supporting Information). Calcium flux full width half max was highest in tissues formed on soft, aligned matrices between soft posts, potentially due to a longer flux plateau^[^ ^7^ ^]^ (Figure S8d,h, Supporting Information). We also treated tissues with the β‐adrenergic agonist isoproterenol to further assess tissue maturation and function (Figure 3k–p). As β‐adrenergic signaling plays a critical role in regulating cardiomyocyte contractility and calcium handling, robust responses to agonists of this pathway, such as increased contractile frequency and stress, are generally indicative of a more mature phenotype.^[^ ^7^, ^18^, ^21^, ^70^ ^]^ Across all conditions tested, tissues demonstrated chronotropic and inotropic responses to isoproterenol (Figure 3k–p; Video S9, Supporting Information). Importantly, isoproterenol induced a greater force‐frequency response in tissues formed on soft (0.68 kPa), aligned matrices with soft (0.41 N m^−1^) posts as compared to tissues formed on stiffer aligned matrices, soft non‐aligned matrices, or stiffer posts (Figure 3k–p).

These findings highlight the value of a bioengineered platform that enables orthogonal tuning of various microenvironmental mechanical inputs, as each input appeared to have unique effects on tissue structure and function. Collectively, the results presented here support the claim that tissues formed on soft, aligned fibrous matrices contracting against soft boundaries resulted in the most structurally and functionally mature EHTs. Additionally, these studies also reveal that each mechanical perturbation impacts tissue function, assembly, and maturation in unique ways. Specifically, reducing fiber alignment impairs both myofibril organization and density resulting in less contractile tissues. Increases in fiber stiffness also yield less dense myofibril networks, promote an arrhythmogenic phenotype, and decrease cell‐matrix adhesions in contrast to findings from studies tuning hydrogel substrate stiffness. Increased post stiffness also resulted in a more prominent arrhythmogenic phenotype, but only when tissues were formed on random matrices between stiff pillars. These studies also showed that combinations of mechanical and architectural inputs can alter tissue structure and function in non‐intuitive ways. For example, while randomly oriented fibers in most combinations of post and fiber stiffness led to decreased tissue contractility and disorganized myofibrils, surprisingly, randomly oriented fibers spanning stiff posts resulted in aligned myofibrils and tissues with heightened contractility. Despite mirroring the highest functioning tissues in terms of contractile stress and myofibril organization, these tissues displayed substantial contractile frequency irregularity consistent with a pro‐arrhythmic phenotype and expressed lower levels of connexin‐43 and MLC‐2v (Figures 2 and 3). Leonard et al. showed that gradually increasing cantilever stiffness yields increases in contractile force to a point where the boundary stiffness reaches potentially pathologic levels.^[^ ^51^ ^]^ Additionally, increased mechanical loading of tissues has been shown to increase myofibril organization,^[^ ^69^, ^71^ ^]^ further supporting the fact that high boundary stiffness may be driving this phenotype. While established EHT platforms have been used to examine the impact of individual mechanical perturbations, we can explore the combinatorial effects of this wide array of physiologically relevant biophysical parameters via the integration of predefined synthetic ECM‐mimetic fibers integrated with microfabricated pillars. Finally, while these studies indicate the importance of control over mechanical features and matrix architecture in driving iPSC‐CM maturation, other maturation techniques such as electrical pacing and metabolic programming are likely essential in deriving tissues that more closely approach the function of healthy adult myocardium.^[^ ^21^, ^72^, ^73^ ^]^ Indeed, culturing fibroTUGs formed with varying post stiffnesses in OxPhos media yielded tissues with greater contractile force after 7 and 14 days in culture as compared to those cultured in standard media^[^ ^18^ ^]^ (Figure S3, Supporting Information). While tissues cultured in baseline RPMI‐1640 medium supplemented with B27 appear to adapt their contractile machinery to exhibit similar levels of force on posts of increasing stiffness, tissues cultured in OxPhos medium for 14 days between 0.9 N m^−1^ posts had the highest contractile function compared to tissues formed between 0.41 and 1.2 N m^−1^ (Figure S3c,d, Supporting Information). These results are consistent with previous studies showing that metabolic maturation can increase the levels of tissue contractile force and further suggest that iPSC‐CM tissues will adapt to increasing boundary stiffness by increasing their contractility until the tissue's boundary stiffness approaches a stiffness regime reflecting pathological conditions.^[^ ^18^, ^23^, ^51^, ^74^ ^]^

### 2.3 Tissue Specific Modeling of fibroTUG Reveals Altered Cellular Response to Matrix Stiffness

**Figure 4:**
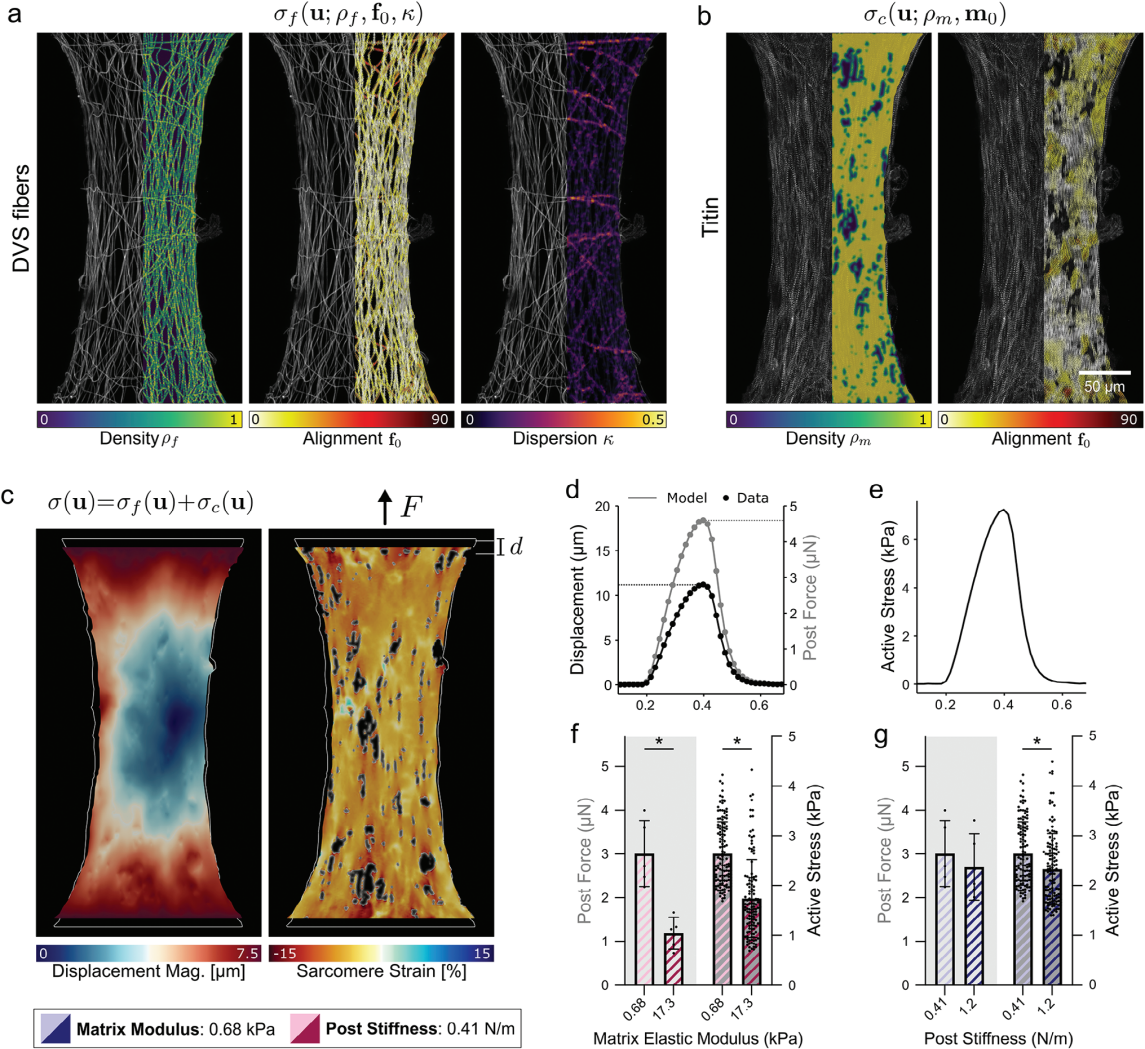
Tissue‐specific computational modeling of fibroTUGs shows altered cellular contractility on matrices of varied stiffness. a) Density, alignment, and dispersion fields characterizing the structure of the fibrous matrix. b) Sarcomere density and alignment characterizing the structure of the myofibril network. c) Results of the simulation for representative tissue showing inner displacement magnitude (left), and sarcomere strain (right). d) Simulated post displacement and force time traces matching the experimental data for one simulation, where post displacement and force data are the simulation input. e) The resulting mean active stress curve exerted by sarcomeres in the model to match the data in (d). f,g) Post‐force input (left two bars on gray background) compared with the computed active stress (right two bars on white background) for n>100 simulations with varied fiber stiffness (f) and varied post‐stiffness (g). All data presented as mean ± std; ^*^ *p* < 0.0001 by unpaired t‐tests.

The ability to orthogonally tune mechanical and architectural inputs to EHT formation enables investigation into how iPSC‐CMs sense and respond to distinct physical microenvironmental cues. However, a limitation of many EHT platforms (including ours) is that tissue‐scale contractile force readouts are determined by measuring post deflections. While this affords a direct measure of dynamic tissue contractility, these measurements fail to capture stresses and strains at the cell and subcellular levels. This is best illustrated by an example of highly contractile CMs contracting on rigid matrices, where limited post deflections would misleadingly suggest low CM contractility. The discrepancy between tissue contractility and cell force/stress thus limits our interpretation of how iPSC‐CMs may be responding to matrix alignment, matrix stiffness, or boundary constraints. To overcome this challenge, we generated tissue‐specific computational models of fibroTUGs that enable quantification of the active cell stresses generated by iPSC‐CMs within these tissues based on the input parameters of composite force, myofibrillar structure, and the underlying fiber structure. Detailed methods on how tissue‐specific models were generated and validated can be found in Jilberto et al.^[^ ^67^ ^]^ Briefly, to generate these computational models, an image analysis pipeline was developed to extract key matrix and cell parameters including fiber density, alignment, and dispersion; sarcomere density and alignment; and tissue displacements (i.e., post deflections) from time‐lapse imaging^[^ ^15^ ^]^ (**Figure** **4**a–c). From these metrics, a non‐linear hyperelastic finite element model that accounts for tissue‐specific fiber and cell mechanics was constructed. The contraction of each tissue was simulated while computing the active stress of CMs required to generate the experimentally determined contractile dynamics (Video S10, Supporting Information). By design, the model captures the contractile behavior experimentally measured at the posts (Figure 4d) and the active stress curve generated by the model describes the heterogenous local contractile function that the CMs generate given the tissue‐specific inputs described above (Figure 4e).

In comparing tissues formed with soft vs. stiff fibers, we found that CMs on stiff matrices in fact generated lower active cell stresses than those on soft matrices (Figure 4f), suggesting that a cellular mechanoresponse to matrix stiffness in part explains the observed reduced force output at the tissue‐scale. The extent to which decreased post‐derived force measurements on stiff matrices are a product of an adaptive cellular response as opposed to the mechanics of the fiber matrices is unknown. However, the magnitude of the decrease in post force on stiff matrices compared to soft was greater than the magnitude of the decrease in the cellular active stress on stiff matrices (Figure 4f), highlighting a combination of cellular mechanosensing, in addition to matrix stiffness and structure, in defining tissue force measured by post deflections. Post stiffness, however, did not significantly change the relationship between tissue and active cell stresses, as expected (Figure 4g). Further exploration of insights gained from the computational model is discussed in the concurrent manuscript.^[^ ^67^ ^]^ Here, we delved deeper into the discussed result and its implications – that matrix stiffness impacts how CMs generate intercellular forces and forces applied extracellularly to the underlying matrix.

### 2.4 Matrix Stiffness Impacts Cell‐ECM Interactions and Costamere Formation

The preceding studies demonstrate that matrix stiffness significantly impacts EHT formation and function (Figures 2 and 3), expression of markers associated with maturation (Figure 3), and active cell stresses (Figure 4). As the fibroTUG platform provides a means to probe how the mechanics of the fibrous ECM impact tissue assembly and maturation, we next examined if cell‐ECM interactions might differ in tissues formed on soft compared to stiff matrices. Previous work from Chopra et al. implicated FAs or protocostameres as critical nucleation points for sarcomere and myofibril assembly in iPSC‐CMs cultured on 2D micropatterns.^[^ ^32^ ^]^ Moreover, Fukuda et al. examined the role of vinculin, a mechanosensitive protein that localizes to cell‐matrix adhesions as well as adherens junctions, in zebrafish heart development.^[^ ^30^ ^]^ Their results indicate that mechanical strain upregulates vinculin expression, which is known to mediate myofibril maturation throughout development. We thus hypothesized that altered cell‐matrix interactions as a function of fibrous matrix stiffness could impact FA and myofibril assembly. Building upon these previous insights and our current findings highlighting changes in iPSC‐CM function and maturation on matrices of varying mechanics, we hypothesized that changes in vinculin localization may coincide with changes in myofibril maturation at later time points (day 7).

**Figure 5:**
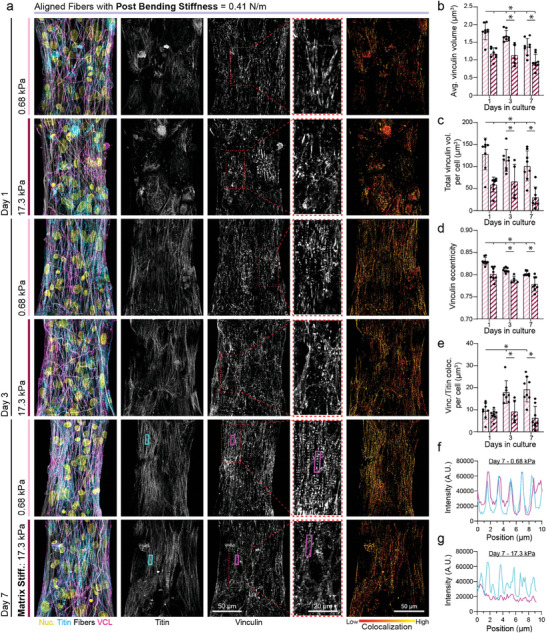
Matrix mechanics influence costamere formation which regulates myofibril assembly and maturation. a) Confocal fluorescent images of fibroTUG tissues fixed at day 1, 3, and 7 post seeding on either soft (0.68 kPa) or stiff (17.1 kPa) aligned fiber matrices (post stiffness was held constant at 0.41 N m^−1^). All images show a region located at the center of each tissue. b) Average vinculin volume, c) total vinculin volume, d) and vinculin eccentricity were quantified from the fluorescent images of immunostained vinculin (n ≥ 5). e) Costamere formation was assessed by quantifying vinculin colocalization with titin. Colocalization of vinculin and titin on day 7 was visualized via fluorescence intensity plots of titin (cyan) and vinculin (magenta) on f) 0.68 kPa matrices and g) 17.1 kPa matrices from lines drawn along the major axis of regions indicated by the rectangles overlayed on images in panel a. All data presented as mean ± std; ^*^ *p* < 0.05.

To test these hypotheses, we generated tissues between soft posts, on aligned soft (0.68 kPa) or stiff (17.3 kPa) matrices and assessed them after 1, 3, or 7 days of culture. Immunostaining for vinculin and quantification of vinculin^+^ adhesion size, shape, and overall abundance via 3D segmentation of confocal z‐stacks revealed marked stiffness‐mediated differences in cell‐matrix interactions during tissue assembly and maturation (**Figure** **5**). At day 1, during initial myofibril assembly, FAs or protocostameres were observed to colocalize with matrix fibers, and the number of vinculin^+^ FAs, average size of FAs, total vinculin expression, and the eccentricity of each FA all were significantly greater on soft matrices as compared to stiff (Figure 5a–e). Further, initially formed, immature myofibrils were more disorganized on stiff matrices than on soft (Figure 3a; Figure S9, Supporting Information). At days 3 and 7, the average vinculin^+^ adhesion size and eccentricity as well as total adhesion volume decreased slightly independent of matrix stiffness (Figure 5c,d). Intriguingly, at these later time points, vinculin localized to z‐discs most prevalently in tissues formed on soft matrices as quantified by colocalization with the z‐disc protein titin. This co‐localization, suggestive of the formation of costameres that tether myofibrils to the matrix, has not to our knowledge been previously reported for iPSC‐CM EHTs (Figure 5a,e‐g). We also noted that on soft matrices, increases in tissue contractility occurring between days 1 and 3 were associated with the enhanced costamere formation that persisted through day 7 (Figure S10, Supporting Information). On stiff matrices however, similar increases in tissue contractile function between day 1 to day 3 appeared to be independent of costamere formation (Figure S10c, Supporting Information). This suggests that improved contractile function during tissue assembly may be mediated by cell‐ECM interactions on soft matrices and by other means, such as the formation of cell‐cell junctions, on stiff matrices.

In native myocardium, costameres physically link myofibrils to the surrounding ECM at each z‐disc, enabling force transmission to adjacent tissue.^[^ ^29^, ^49^, ^50^ ^]^ These structures are known to play a critical role in regulating myocardial contractile function and their formation during development may be regulated in part by mechanical strain within the tissue.^[^ ^30^, ^31^ ^]^ We also observed an increase over time in the expression of the β1D integrin splice isoform uniquely in soft matrices (Figure S11, Supporting Information). Integrin β1D is specific to cardiac and striated muscle cells and has previously been associated with cardiac maturation.^[^ ^75^, ^76^, ^77^ ^]^ Taken together, the formation of costameres and increased expression of β1D integrin may indicate a more mature CM adhesive state regulated by matrix mechanics that corresponds to the formation of more mature myofibrils (Figure 3c,d).

To further explore the connection between tissue maturation and costamere formation, we cultured fibroTUG tissues generated on soft, aligned matrices spanning soft posts in OxPhos media for 21 days to test the potential for further iPSC‐CM maturation.^[^ ^18^, ^73^ ^]^ Unlike previously established models that rely on 2D micropatterning,^[^ ^18^ ^]^ the formation of robust cell‐ECM adhesions and long‐term stability of fibrous DVS matrices used in this platform facilitates the investigation of relationships between tissue maturation and cell‐ECM interactions over long‐term culture. Fixing and imaging tissues immunostained for vinculin and GFP‐titin at days 1, 3, 7, 14, and 21 revealed that vinculin colocalization with the z‐disc persists at later time points as the tissues continue to mature (Figures S12a,e,f, and S5, Supporting Information). As was the case in tissues cultured in standard RPMI B27 media, FA average size trended downward over time as did adhesion eccentricity, supportive of a transition in CM adhesion from FAs or protocostameres to costameres (Figure S12b,d, Supporting Information). However, the total volume of vinculin‐enriched structures in the tissue increased at later time points, suggesting continued remodeling of cell‐matrix adhesions in conditions that drive tissue maturation (Figure S12d, Supporting Information). As previously described, costameres are composed of various proteins that play specific roles in processes such as cellular mechanosensing and signal transduction. We thus confirmed our previous findings by additionally immunostaining for zyxin, an adhesion protein associated with adhesion maturation that regulates actin polymerization.^[^ ^38^, ^78^, ^79^ ^]^ In contrast to vinculin, average zyxin volume remained relatively constant while total zyxin expression and eccentricity increased over time (Figure S13a–d, Supporting Information). Additionally, zyxin localization to costameres (based on co‐localization with α‐actinin) lagged behind vinculin, peaking at day 14 as opposed to day 7 (Figure S13a,e,f, Supporting Information). This is consistent with the notion that zyxin is recruited to more mature adhesions based on previous observations of zyxin recruitment to adhesions following early recruitment of vinculin and paxillin.^[^ ^80^, ^81^ ^]^

**Figure 6:**
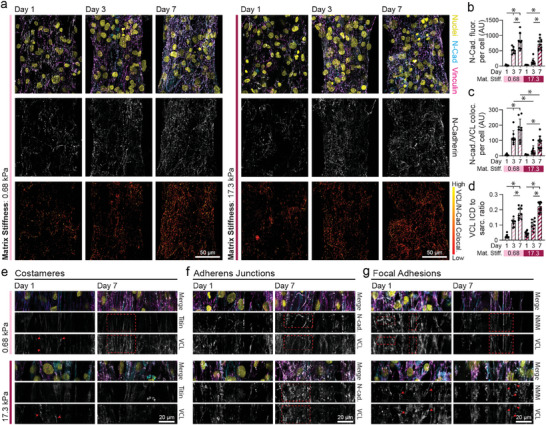
Microenvironmental mechanics regulate vinculin localization to costameres, adherens junctions, and focal adhesions. a) Confocal fluorescent images of fibroTUG tissues fixed at days 1, 3, and 7 after seeding on either soft (0.68 kPa) or stiff (17.1 kPa) aligned fiber matrices (post stiffness was held constant at 0.41 N m^−1^). All images show a region located at the center of each tissue. b) N‐cadherin (N‐Cad) fluorescence was quantified from the fluorescent images of immunostained N‐cadherin (n ≥ 8). c) Vinculin localization to adherens junctions was quantified by analyzing vinculin and N‐cadherin immunostained images. d) Ratio of vinculin that localized to intercalated discs (ICD) to vinculin localized to costameres or sarcomere z‐discs. Representative confocal fluorescent images of vinculin localization at days 1 or 7 to e) costameres (i.e., vinculin colocalization to titin), f) adherens junctions (i.e., vinculin colocalization to N‐cadherin), and g) focal adhesions (i.e., vinculin colocalization to NMM‐IIB) not associated with either N‐cadherin or titin staining, as indicated by red arrow heads and boxes. All data presented as mean ± std; ^*^ *p* < 0.05.

As vinculin also localizes to adherens junctions, we co‐immunostained tissues fixed at days 1, 3, and 7 for N‐cadherin and vinculin to examine whether N‐cadherin (N‐cad) and vinculin co‐localization at the ICD was also influenced by matrix stiffness (**Figure** **6**a–d). Total N‐cadherin expression increased comparably over culture time in tissues formed on both soft and stiff matrices (Figure 6a,b). Expression of desmoplakin, a key desmosomal protein which also localizes to the ICD, also increased similarly with time on soft and stiff matrices (Figure S14, Supporting Information). Localization of vinculin to N‐cadherin, however, was greatest on soft matrices at days 3 and 7, potentially indicating the formation of more robust and mechanically engaged ICDs (Figure 6a,c). When normalizing the intensity of vinculin at ICDs to costameric vinculin, differences between the two matrix conditions were not apparent, indicating that vinculin expression is upregulated in both locations in the more contractile tissues formed on soft matrices (Figure 6d). This idea is supported by findings from Fukuda et al. that indicate vinculin localization to both costameres and ICDs is upregulated in CMs that experience mechanical strain during development.^[^ ^30^ ^]^

Using high resolution imaging, we identified three distinct locations to which vinculin localizes in fibroTUG tissues: 1) FAs or protocostameres, most prevalent upon initial cell adhesion and during myofibril assembly (day 1), 2) ICDs, and 3) costameres, which were evident by day 7 following myofibril formation^[^ ^49^ ^]^ (Figure 6e–g; Figure S15, Supporting Information). Of note, vinculin localized to z‐discs to form costameres preferentially in soft matrices by day 7, suggesting myofibril maturation may be associated with the formation of these critical cell‐ECM adhesions (Figure 6e). Vinculin was present at N‐cadherin‐rich adherens junctions in both soft and stiff matrices by day 7 (Figure 6f; Figure S15, Supporting Information), despite the lower amount of N‐cadherin colocalization with vinculin observed in stiff tissues (Figure 6c). Finally, we observed vinculin localization to FAs distinct from z‐discs based on the lack of titin and instead co‐localization with non‐muscle myosin‐IIB (NMM‐IIB) (Figure 6g). FAs were particularly prominent in assembling tissues on day 1 and more commonly observed on stiff matrices across all time points (Figure 6g), in line with decreased vinculin localization to titin noted on stiff matrices (Figure 5e). Furthermore, these complexes were larger and more elongated on soft matrices than stiff matrices at day 1, suggesting more robust adhesion to soft matrices, as previously described (Figure 6g and 5d). These three vinculin‐enriched adhesive structures were first observed by Simpson and colleagues in adult feline CM cultures.^[^ ^49^ ^]^ Their results suggest that contractile behavior and the formation of cell‐cell junctions regulate vinculin distribution and potentially myofibril assembly.^[^ ^32^, ^46^, ^49^, ^50^ ^]^ Our observations indicate that cellular mechanosensing of the ECM drives the expression and localization of vinculin to distinct cellular domains within iPSC‐CMs and further supports the role that contractile activity plays in regulating vinculin distribution (Figures 5 and 6).

### 2.5 Tissue Contractility Drives Myofibril Maturation and Costamere Formation in Soft Matrices

**Figure 7:**
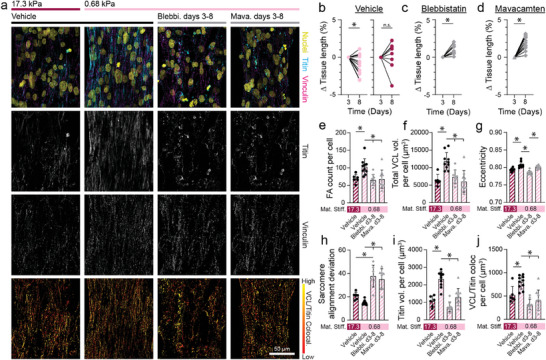
Tissue contractility drives the maturation and maintenance of myofibrils and costameres. a) Confocal fluorescent images of fibroTUG tissues treated with blebbistatin (50 µm) or mavacamten (500 nM). All images show a region located at the center of each tissue. b) Diastolic tissue length on days 3 and 8 of tissues seeded on soft (0.68 kPa) and stiff (17.1 kPa) aligned fiber matrices (post stiffness was held constant at 0.41 N m^−1^) without treatment with the contractile inhibitors. c,d) Diastolic tissue length of tissues seeded on soft matrices on day 3 before treatment with contractile inhibitors, (c) blebbistatin or (d) mavacamten, and day 8 after 5 days of treatment (n ≥ 8). e) Focal adhesion count, f) vinculin volume per cell, and g) focal adhesion eccentricity were quantified from the fluorescent images of immunostained vinculin (n ≥ 6). h) Sarcomere alignment deviation and i) titin volume per cell quantified from fluorescent images of titin‐GFP reporter (n ≥ 6). Vinculin colocalization with titin per cell quantified from titin and vinculin images (n ≥ 6). All data presented as mean ± std; ^*^ *p* < 0.05.

In the preceding studies, soft (0.68 kPa), aligned fibrous matrices yielded the most contractile tissues and localization of vinculin to costameres, an adhesive phenotype not previously reported in EHTs derived from iPSC‐CMs (Figures 2,5,6). As prior studies implicated myosin contractility as being critical for myofibril assembly and maturation,^[^ ^18^, ^32^, ^36^, ^82^ ^]^ we next examined if myosin contractility also plays a role in regulating myofibril stability and costamere formation. iPSC‐CMs seeded on soft matrices were treated with blebbistatin (50 µM) beginning at day 3, an inhibitor of both non‐muscle myosins and cardiac myosins, or mavacamten (500 nM), a cardiac myosin specific inhibitor, to test whether myosin contractility is required for myofibril maturation and concomitant vinculin localization to costameres (**Figure** **7**). Comparisons were made to tissues formed on stiff matrix conditions, which exhibited less costameric vinculin and overall lower myofibril density (Figure 5). Tissues were analyzed on day 8 to assess diastolic stress, vinculin localization, and myofibril assembly. In only untreated tissues on soft matrices, we observed a decrease in diastolic tissue length implying enhanced tissue contractility (Figure 7b). In contrast, blebbistatin and mavacamten treated tissues both increased in length, indicating relaxation and lower diastolic stress (Figure 7c,d). Further, the number of vinculin^+^ adhesions, total vinculin expression, and eccentricity of adhesive structures decreased in tissues treated with both blebbistatin and mavacamten from days 3–8, implying disrupted maturation of cell‐cell and cell‐ECM adhesions (Figure 7e–g). Adhesion organization and vinculin localization in treated tissues formed on soft matrices were similar to that of untreated stiff matrix tissues, suggesting that diminished CM contractility on stiff matrices may limit adhesion maturation (Figure 7e–g). No effect was observed when treating tissues from only days 7–8, most likely due to the shorter treatment duration that was not long enough to allow significant disassembly of cell‐ECM adhesions and myofibrils (Figure S16, Supporting Information). Additionally, myofibril organization and density, along with non‐muscle myosin IIB expression, decreased upon myosin inhibition, supporting a role for actomyosin contractility in myofibril maintenance (Figure 7h,i; Figure S17, Supporting Information). Finally, vinculin localization to z‐discs decreased in treated tissues, implying that actomyosin contractility is critical for the formation of costameres (Figure 7j).

Variants in β‐cardiac myosin (MYH7) and other regulators of contractility are associated with hypertrophic cardiomyopathies (HCM) and dilated cardiomyopathies (DCM). In fact, nearly a third of all known HCM variants arise from mutations in MYH7, in line with our findings that myosin driven CM contractility is critical for myofibril assembly, maturation, and overall structural integrity.^[^ ^43^, ^44^ ^]^ Additionally, many costameric proteins including vinculin and filamin C, are strongly implicated in dilated cardiomyopathies.^[^ ^43^, ^83^, ^84^ ^]^ Studying the impact of these mutations on tissue function requires accurate models of the myocardium that possess adult‐like function and structure.^[^ ^7^ ^]^ The fibroTUG platform provides the mechanical control and ECM‐like architecture of fibrous matrices necessary for driving a mature cell‐adhesive phenotype that may be critical to a deeper understanding of the mechanisms of such diseases.

### 2.6 Costamere Formation is Associated with Mature Myofibrils

**Figure 8:**
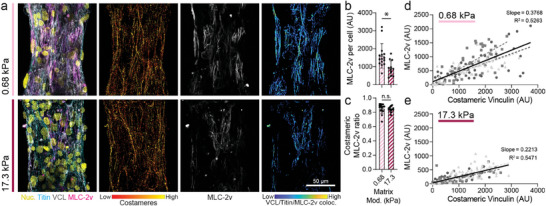
Costamere formation is associated with more mature myofibrils. a) Confocal fluorescent images of fibroTUG tissues fixed at day 7 after seeding on either soft (0.68 kPa) or stiff (17.1 kPa) aligned fiber matrices (post stiffness was held constant at 0.41 N m^−1^). b) Quantification of MLC‐2v expression per cell (n ≥ 12). c) Ratio of MLC‐2v expressing myofibrils that are associated with robust costamere formation (n ≥ 12). For tissues formed on d) soft and e) stiff matrices, the correlation between MLC‐2v expression and costameric vinculin was assessed by segmenting tissues into regions equal to roughly the size of one CM and MLC‐2v and costameric vinculin expression was quantified within each of these regions. Each point on the plot represents a single subregion on one of three representative tissues. Linear regressions for data from each individual tissue are indicated by different colored dashed lines while linear regression of the data from these three tissues pooled together is indicated by the solid black line. The slope and R^2^ values for this pooled data are also noted in the plots. All data presented as mean ± std; ^*^ *p* < 0.05.

As costameres are the sole mediator of CM‐ECM adhesion in the mature adult myocardium, we next tested the hypothesis that MLC‐2v expression directly correlates with costamere formation (**Figure** **8**). Shared expression of costameric vinculin and MLC‐2v could suggest that robust cell adhesion to the matrix along the myofibril facilitates myofibril maturation via recruitment of the more mature myosin light chain isoform. We seeded iPSC‐CMs containing a GFP‐titin reporter on both soft and stiff aligned matrices tethered between soft posts and immunostained for MLC‐2v and vinculin after 7 days of culture (Figure 8). As before, we observe increased MLC‐2v expression and vinculin localization to z‐discs on soft matrices. In contrast, iPSC‐CMs on stiff matrices revealed significantly lower MLC‐2v expression (Figure 8a,b). Interestingly, examining the ratio of MLC‐2v colocalized with vinculin‐rich costameres, we found that the percentage is high in both soft and stiff matrices, despite decreased overall expression of MLC‐2v in stiff matrices, indicating a relationship between myofibril maturation and costamere formation (Figure 8c). To support this observation, we segmented the tissue into subsections comparable to the area inhabited by a single spread CM. For each of these subregions, MLC‐2v expression was plotted against costameric vinculin and fit to a linear regression model (Figure 8d,e). On both soft and stiff matrices, the correlation between these two metrics was positive and significant; however, a higher linear regression slope was noted for tissues formed on soft matrices, indicating that the highest level of both costameric vinculin MLC‐2v was attained in tissues on soft matrices (Figure 8d,e). Taken together, these data indicate that the formation of costameres is coupled with myofibril maturation, though costamere formation does not necessarily lead to myofibrillar maturation. This finding highlights the critical relationship between CM cell‐matrix adhesion and myofibril maturation that hand in hand define CM and overall tissue contractility.

## 3 Discussion

Despite many recent advances, studying the impact of biophysical cues on iPSC‐CM tissue assembly and maturation remains challenging. Current 2D models provide an excellent setting for certain applications, but typically cannot recapitulate native tissue mechanics and architecture. Common 2D culture platforms such as micropatterned ECM proteins, for example, lack the fibrous topography and mechanical heterogeneity present in native myocardial tissues.^[^ ^17^, ^18^, ^26^ ^]^ Electrospun scaffolds have limited mechanical control, as materials such as polycaprolactone are much stiffer than fibrillar proteins in the ECM and lack mechanical tunability.^[^ ^16^, ^25^, ^85^ ^]^ Additionally, while widely used 3D constructs more closely mimic the dimensionality of native microenvironmental conditions and enable control of some relevant mechanical inputs such as boundary stiffness, isolating the impact of tissue‐relevant mechanical and architectural cues on CMs in these models is intractable due to the use of natural biomaterials.^[^ ^8^, ^9^ ^]^ Thus, new EHT platforms that capture the fibrous architecture of the native ECM while enabling orthogonal tuning of boundary stiffness, matrix alignment, and stiffness (over a physiologic range) may provide new insights into iPSC‐CM mechanosensing, function, and structural maturation. Thus, we created a 2.5D tunable iPSC‐CM tissue platform composed of mechanically tunable synthetic fiber matrices suspended between two elastomeric posts, enabling the study of key physical microenvironmental inputs to tissue assembly and function: tissue boundary constraints, matrix fiber alignment, and matrix stiffness.

Unlike other approaches to engineering cardiac tissues, the orthogonal tunability of the fibroTUG cardiac microtissue platform allowed for careful dissection of how individual biophysical cues impact CM organization and function. While several studies have examined how mechanical inputs impact iPSC‐CM function and tissue assembly, previously established platforms lack the ability to isolate the effects of relevant, distinct physical properties in a setting that faithfully recapitulates the architecture of the myocardial microenvironment. Furthermore, the formation of thin fibroTUG tissues (≈15 µm thick) between elastomeric posts permits comprehensive image‐based analysis of both contractile function and subcellular structural development of the tissues, which enabled insights into the distinct impacts each mechanical input has on tissue assembly, contractility, and maturation.

Each mechanical perturbation made possible by the fibroTUG platform corresponds to various microenvironmental states that occur in native tissue development and disease progression. Our studies exploring the influence of fiber stiffness provide insight into how changes in ECM mechanics during cardiac development or disease progression may impact cardiac tissue organization and function. Alternatively, matrix alignment is known to play a critical role in tissue function, where disorganized fibrotic ECM deposited following myocardial injury can contribute to abnormal myocardial biomechanics and disease‐associated signaling.^[^ ^10^, ^86^ ^]^ Finally, control over post stiffness to define tissue boundary constraints may reflect tissue remodeling associated with altered cardiac afterload, which is known to alter heart function in various contexts.^[^ ^44^, ^51^, ^69^, ^71^, ^87^ ^]^

Importantly, many cardiac microtissue platforms require the inclusion of stromal cells for tissue assembly and compaction.^[^ ^8^, ^21^, ^22^, ^23^, ^24^, ^88^, ^89^ ^]^ However, their random distribution amongst contractile CMs does not accurately recapitulate the organization of the native myocardium where CMs and stromal cells are segregated,^[^ ^10^, ^11^, ^13^ ^]^ and furthermore confounds clear insights into how various biophysical cues impact specifically CM development and maturation. Additionally, these tissues typically exhibit limited stability over time in culture due to stromal cell proliferation, contraction, and tissue delamination.^[^ ^7^, ^8^ ^]^ In our fibroTUG platform, predefining matrix properties before seeding enables the assembly of robust iPSC‐CM tissues without the addition of stromal cells. There is mounting evidence to suggest that CM‐fibroblast crosstalk via both direct cell‐cell connections and paracrine signaling can drive iPSC‐CM maturation.^[^ ^89^, ^90^ ^]^ Expanding on this idea, these studies show that, independent of other biochemical signals, CM mechanosensing of synthetic matrices that recapitulate fibroblast secreted perimysial collagen fibers can significantly impact CM development and function. Future studies using this platform will be focused on comparing tissues formed from pure iPSC‐CM populations to those containing fibroblasts to probe how fibroblasts might impact iPSC‐CM maturation.

We found that tissues formed on soft (0.68 kPa), aligned matrices suspended between soft (0.41 N m^−1^) posts were the most mature, as evidenced by myofibril density and contractility (Figures 2 and 3). These optimized mechanical conditions yielded tissues with comparable contractile stress output to tissues generated in other studies.^[^ ^21^, ^51^, ^69^, ^91^, ^92^, ^93^, ^94^ ^]^ However, most of these approaches yield tissues that were much larger and therefore composed of substantially more CMs (often >1e6 CMs) as compared to our tissues composed of an average of ≈100 CMs. Further, fractional shortening of fibroTUG tissues was maximally ≈5% compared to slightly higher values reported in some 3D in vitro model constructs that report ≈10% fractional shortening,^[^ ^51^, ^69^ ^]^ both of which fall short of values reported for the left ventricular myocardium (≈20%).^[^ ^95^ ^]^ Nonetheless, our fibroTUG microtissues contract with higher stress than myocardial tissue strips obtained from human newborn and infant hearts (≈0.9 kPa).^[^ ^96^ ^]^ Taken together, this mechanically tunable platform can drive the formation of functional iPSC‐CM tissues.

Each mechanical input we explored had distinct effects on tissue assembly, maturation, and contractility, suggesting that different mechanosensing mechanisms may be involved in responding to various mechanical signals. First, altered contractile function of tissues formed in aligned vs. randomly oriented fiber matrices is likely a result of disrupted myofibril density and organization. (Figure 2). By isolating matrix alignment from matrix and boundary stiffness perturbations, we can conclude that iPSC‐CM interactions with disorganized matrices lead to impaired formation of aligned myofibrils. Specifically, we found that increased fiber alignment improved myofibril alignment and density, independent of fiber stiffness and post stiffness. We also observed that matrix alignment enhances tissue contractile function and calcium handling, as has been previously described.^[^ ^15^, ^16^, ^17^, ^18^, ^97^ ^]^ Second, isolating the impact of altered matrix stiffness in our tissue platform revealed that the contractility of CMs on soft vs. stiff fiber matrices is in part defined by differential mechanosensing and CM‐matrix interactions. Using non‐degradable, mechanically defined, and well‐characterized synthetic fibers that can be easily imaged facilitated the creation of tissue‐specific modeling the provided precise analysis of how matrix architecture and mechanics impact iPSC‐CM contractile function.^[^ ^67^ ^]^ This integration of finite element models of our fibroTUG platform provided further insights into how iPSC‐CMs respond to this specific mechanical perturbation and confirmed that CMs on stiff matrices indeed individually generate less stress (Figure 4). Specifically, we found that differences in tissue contractility as measured by elastomeric posts were not only caused by changes in matrix deformability but also a cellular contractile response to changes in matrix stiffness, as measured by changes in cellular active stresses (Figure 4). Further supporting this claim, cell adhesions, and more specifically the formation of costameres, proved sensitive to matrix stiffness, implying that cellular mechanosensing dictates the formation of distinct adhesive structures that may influence tissue assembly and structural maturation (Figure 5). Current computational modeling efforts are focused on integrating cell‐ECM and cell‐cell adhesions into the system with the goal of better understanding how tissue mechanosensing impacts tissue growth or disease progression.^[^ ^67^ ^]^ Lastly, while increasing post stiffness did not impact cellular active stress or post‐force measurements, stiff boundary constraints appear to be sufficient in driving myofibril alignment regardless of matrix organization, despite low levels of fractional shortening.

Fiber alignment plays a key role in the development of fibroTUG tissues, and previously developed techniques including electrospun scaffolds, nano‐ and micro‐grooved surfaces, or micropatterning have been shown to drive alignment and subsequent maturation of iPSC‐CMs.^[^ ^15^, ^16^, ^17^, ^18^, ^19^ ^]^ Additionally, many groups have explored how matrix stiffness impacts CM maturation and contractile function, culminating in the observation that CMs contract most robustly on hydrogels of physiologic stiffness (≈8‐10 kPa).^[^ ^17^, ^54^ ^]^ In our studies, soft, fibrous matrices (0.68 kPa) that more closely mimic the stiffness of fetal heart tissue yielded the most contractile tissues.^[^ ^54^ ^]^ This discrepancy could be explained by the distinct architecture and local mechanics of fibrous matrices as compared to isotropic, continuum‐like hydrogel or elastomer surfaces that lack discrete fibrous structure and topography.^[^ ^15^ ^]^ In addition to better modeling CM responses in the native tissue microenvironment, this tissue model may also better mimic the mechanics of other 3D EHT constructs made from fibrous hydrogels such as fibrin or collagen. Furthermore, in our platform, tissues formed on soft, aligned matrices with soft boundary conditions displayed the highest levels of fractional shortening, a key regulator of iPSC‐CM maturation.^[^ ^18^, ^69^, ^92^ ^]^ Caution should be taken, however, when attempting to compare elastic moduli of hydrogels and other in vitro culture platforms with the modulus of native tissues, as characterization techniques vary widely across settings (eg., tested in compression versus tension, or at different length‐scales). Additionally, as highlighted here, cells sense and respond to many mechanical properties of a material that are not captured in a simple elastic modulus measurement. Previous work from our group showcases this idea, demonstrating that fibroblasts or mesenchymal stem cells cultured on fibrous scaffolds generate more robust focal adhesions when individual fibers and resulting matrices are softer rather than stiffer, in contrast to previous studies showing great focal adhesions formation on stiffer elastic hydrogel or elastomer surfaces.^[^ ^52^, ^55^ ^]^ As it is well accepted that iPSC‐CMs are most comparable to fetal CMs, the results presented here support the concept of initiating CMs in a soft environment to allow them to assemble properly prior to gradually increasing matrix mechanics to promote CM growth and maturation, as has been previously explored using elastic hydrogels.^[^ ^53^ ^]^

One aspect of this mechanoresponse involves the formation and modulation of costameres. In agreement with previous work,^[^ ^32^, ^36^ ^]^ we observed that protocostamere formation corresponds with initial myofibril assembly. However, our experiments examined time points beyond the early spreading of iPSC‐CMs mediated by protocostameres in a multicellular, tissue‐like context. Intriguingly, we found that costamere formation, as evidenced by vinculin and zyxin localization to the z‐disc, is maximized when tissues are maintained on soft (0.68 kPa) fiber matrices (Figure 5). Previous results obtained by culturing single iPSC‐CMs on soft versus stiff hydrogels indicated that the stiffness of the hydrogel surface did not impact costamere formation, supporting the idea that iPSC‐CMs interact differently with fibrous matrices compared to isotropic hydrogel surfaces.^[^ ^38^ ^]^ Further, these findings support a dynamic interplay between adhesion myofibrillar proteins where protocostameres give rise to myofibrils followed by a redistribution of adhesion proteins to along the myofibril under particular mechanical conditions. Additionally, we found that iPSC‐CMs possessing more costameres also demonstrated increased expression of MLC‐2v, a marker of mature myofibrils in ventricular CMs (Figure 8). As costameres play a critical role in transmitting force generated by the myofibril to the surrounding matrix, these results suggest that mechanical inputs from the microenvironment influence the formation of distinct types of matrix adhesions and myofibrillar content. Despite observing that MLC‐2v expression and costameres correlate on a per cell basis (Figure 8), the connection between the two remains to be explored.

Tissues formed on soft, aligned matrices were shown to have the highest contractile function (Figures 2 and 5; Figure S10, Supporting Information), robust costamere formation, and a heightened density and alignment of myofibrils, but the temporal relationship between these phenotypes is unknown. Fukuda et al. show that increases in cardiac contractility in the developing zebrafish heart correlates to enhanced vinculin localization to adhesions, which appears to be critical for myofibril maturation.^[^ ^30^ ^]^ As there are clear correlations between contractility, cell‐ECM adhesion maturity, and myofibril development in our iPSC‐CM‐based tissues, mechanistic studies aimed at parsing the specifics of these relationships would help advance our understanding of the molecular mechanisms regulating costamere formation and their connection to iPSC‐CM maturation. The interactions of various focal adhesion proteins such as vinculin, zyxin, talin, paxillin, focal adhesion kinase, and others as adhesions mature have been explored extensively in other cell types such as fibroblasts.^[^ ^98^ ^]^ CMs experience different magnitudes and dynamics of mechanical loading compared to these cell types, so exploring the complex relationships between the many proteins involved in costamere formation in iPSC‐CMs will be critical to understanding their role in CM maturation.

Our results may also inform the design of larger‐scale tissue patches and translatable regenerative therapies, where interactions between iPSC‐CMs and biomaterials are likely critical to the proper assembly of functional, mature myocardial syncytia. Future work translating the identified optimal mechanical parameters to fully 3D tissue constructs with therapeutic potential will be essential. Previous work developing 3D EHT constructs lacked close examination of cell‐ECM interactions, at least in part due to imaging challenges inherent to these thick tissue constructs. However, it is critical to understand how CMs interact with their microenvironment in these constructs, as focal adhesions and costameres are likely required for not only native tissue‐like contractile function of EHTs but also robust integration of EHTs with native tissue upon implantation due to their critical roles in force transmission. Scaffold design features such as the incorporation of soft, aligned fibers should be considered, especially in light of prior studies testing scaffold‐free implants which resulted in arrhythmic activity.^[^ ^99^, ^100^ ^]^ Motivated by the findings in this work, we are currently working to integrate synthetic fibers into fibrin‐ or collagen‐based 3D iPSC‐CM tissue constructs to examine whether findings presented in these studies translate to the biofabrication of larger scale tissue patches.

Furthermore, as alterations in myocardial matrix organization and mechanics are a hallmark of many forms of cardiac disease, the high mechanical tunability of the fibroTUG platform could enable key insights into the mechanisms of disease and in the longer term, facilitate the development of better treatment options. Of note, mutations in mechanosensitive proteins, including vinculin, have been shown to cause various forms of heart disease such as dilated or hypertrophic cardiomyopathies.^[^ ^43^, ^44^ ^]^ Ongoing studies are being conducted to better understand how changes in tissue mechanics may exacerbate disease phenotypes observed in patients with genetic cardiomyopathies, specifically in proteins of the costamere or intercalated disc.^[^ ^34^, ^43^, ^44^ ^]^ Finally, in vitro CM tissue models, such as the fibroTUG platform presented here show promise as platforms for screening drugs for potential cardiotoxicity or effective in treating heart disease. Here, we show that tissue response to isoproterenol, a clinically approved inotrope used to treat patients with heart failure, is impacted by altered tissue mechanics, highlighting a requirement for the informed design of drug screening platforms (Figure 3 k‐p).

In conclusion, here we developed a mechanically tunable iPSC‐CM microtissue platform that we used to investigate the impacts of physiologically relevant microenvironmental cues on EHT formation, function, and maturation. This and similar platforms can help drive progress in the field of cardiac tissue engineering by providing mechanistic insights into how CMs interact with their native ECM or engineered scaffolds towards generating better in vitro tissue models of disease or developing tissue‐replacement therapies.

## 4 Experimental Section

### Reagents

All reagents were purchased from Sigma–Aldrich and used as received unless otherwise stated.

### Elastomeric Cantilever Array Fabrication

Arrays of poly(dimethylsiloxane) (PDMS; Dow Silicones Corporation, Midland, MI) posts were fabricated by soft lithography as previously described [cite DexVS paper and bdon nat mat]. Briefly, silicon wafer masters possessing SU‐8 photoresist (Microchem, Westborough, MA) were produced by standard photolithography and used to generate PDMS stamps. Following silanization with trichloro(1H,1H,2H,2H‐perfluorooctyl)silane, stamps were used to emboss uncured PDMS onto oxygen plasma‐treated coverslips. Cantilever arrays were methacrylated with vapor‐phase silanization of 3‐(trimethoxysilyl)propyl methacrylate in a vacuum oven at 60 °C for at least 6 h to promote fiber adhesion to PDMS.

### DVS Fiber Matrix Fabrication

DVS polymer was synthesized as previously described by the lab.^[^ ^52^ ^]^ Briefly, dextran was reacted with divinyl sulfone, and the product was dialyzed and lyophilized. For electrospinning, DVS was dissolved at 0.7 g mL^−1^ in a 1:1 mixture of milli‐Q water and dimethylformamide with 0.6% (w/v) lithium phenyl‐2,4,6‐trimethylbenzoylphosphinate (LAP; Colorado Photopolymer Solutions) photoinitiator, 2.5% (v/v) methacrylated rhodamine (25 mM; Polysciences, Inc., Warrington, PA), and 5.0% (v/v) glycidyl methacrylate. This solution was electrospun on coverslips containing microfabricated cantilever arrays affixed to a custom‐built rotating mandrel with a hexagonal geometry driven by an AC motor with controllable speed.^[^ ^15^ ^]^ Electrospinning was conducted in an environmental chamber at 35% humidity with a flow rate of 0.2 ml h^−1^, voltage of 7.0 kV, and a gap distance of ≈5 cm to the grounded mandrel. After collection, fibers were stabilized by primary crosslinking under UV (100 mW cm^−2^) through a microfabricated photomask for 20 s, such that only the fibers suspended in the region spanning two posts would be crosslinked. Upon hydration, uncrosslinked fibers were dissolved away leaving isolated suspended microtissues adhered to the posts. Fiber matrices were subsequently placed in LAP photoinitiator solutions of varying concentrations and exposed again to UV (100 mW cm^−2^) for 20 s to tune fiber stiffness and sterilize substrates.

Matrices were functionalized with cell adhesive peptides cyclized [Arg‐Gly‐Asp‐D‐Phe‐Lys(Cys)] (cRGD; Peptides International) via Michael‐Type addition to available vinyl sulfone groups. Peptides were dissolved at 200 µM in milli‐Q water containing HEPES (50 mM), phenol red (10 µg mL^−1^), and 1 m NaOH to bring the pH to 8.0. A volume of 150 µL was added to each substrate and incubated at room temperature for 30 min.

### Mechanical Characterization

PDMS cantilever mechanics were characterized by deflecting individual posts with a ≈100 µm diameter tungsten rod of known elastic modulus attached to a micromanipulator (SmarAct) (Figure S1a–c, Supporting Information). Bending stiffness was calculated by measuring the cantilever deflection and the force applied by the tungsten rod using custom Matlab scripts. Bending stiffness was approximated using the following equations:

(1) $$F_{rod}=\frac{3dEI_{rod}}{L^{3}}\;I_{rod}=\frac{\pi r^{4}}{4}\;Bending\;stiffness\;\;\left(k\right)=\;\;\frac{F_{rod}}{\delta }$$

where d = rod deflection, E = elastic modulus of rod, L = length of the rod, r = radius of the rod, δ = post deflection, F_rod_ = force applied by the rod, and I_rod_ = moment of inertia of the rod. Rod deflection (d) was quantified by subtracting the distance that the PDMS post moved (δ) from the distance moved by the micromanipulator holding the rod.

Matrix modulus was determined by pressing a microfabricated SU8 rectangle measuring 20 × 250 µm across the center of the fiber matrices to apply tension to the matrix (Figure S1d–f, Supporting Information). Using custom Matlab scripts, the matrix modulus was extrapolated from the applied force (measured by the PDMS post's deflection) and the resulting tensile stretch of the fiber matrix. The matrix modulus was approximated using the following equations. First, the resultant length of half of the stretched fiber matrix L_f_ was geometrically defined as:

(2) $$L_{f}=\sqrt{{\left(\Delta h\right)}^{2}+{\left(\frac{L_{0}}{2}-\delta \right)}^{2}}$$

where Δ*h* = indentation depth, *L*_0_ = initial length of the fiber matrix, and δ = post deflection. As the resultant post deflection is the result of a force balance between the cantilever and resistance arising from tension in the stretched fiber matrix, the force was defined on each post *F*_*p*_ as:

(3) $$F_{p}=T\;sin\theta$$

where *T* = tension in the fiber matrix and θ = angle between the indenter and the fiber matrix. Tension in the matrix can thus be defined as follows:

(4) $$T=F_{p}\frac{L_{f}}{\frac{L_{0}}{2}-\delta }$$

Next, the elastic modulus was defined of the fiber matrix, $\;E=\frac{\sigma }{\varepsilon }$, in terms of the measured parameters, where σ = stress and ε = strain.

(5) $$\sigma =\frac{T}{A}=\frac{F_{p}L_{f}}{A\left(\frac{L_{0}}{2}-\delta \right)}$$

(6) $$\varepsilon =\frac{2L_{f}-L_{0}}{L_{0}}$$

(7) $$E=\frac{TL_{0}}{A\left(2L_{f}-L_{0}\right)}$$

More details on this calculation and mechanical testing set up can be found in Figure S1 (Supporting Information).

### iPSC Culture and iPSC‐CM Differentiation

Induced pluripotent stem cells containing a GFP‐titin reporter^[^ ^101^ ^]^ (PGP1; gift from the Seidman Lab) or GFP‐DSP reporter (WTC; Allen Institute AICS‐0017 cl.6) were cultured in mTeSR1 media (StemCell Technologies) on Matrigel (Corning) coated tissue culture plastic and differentiated via temporal Wnt modulation as previously described.^[^ ^4^, ^5^ ^]^ Briefly, differentiation was initiated when iPSCs reached 90% confluency in RPMI 1640 media supplemented with B27 minus insulin on day 0 with the addition of 12 µm CHIR99021 for 24 h. On day 3, CDM3 media containing 5 um IWP4 on day 3 for 48 h. Retinol inhibitor BMS 453 (Cayman Chemical, 1 µm) was also added for days 3–6 to minimize atrial lineage differentiation.^[^ ^18^, ^102^ ^]^ Cultures were then maintained in CDM3 media until contractions began between days 8 and 10. iPSC‐CMs cultures were then transferred to RPMI 1640 media lacking glucose and glutamine (Captivate Bio) supplemented with 4 mM DL‐lactate, 500 ug mL^−1^ human serum albumin (Sciencell Research Labs), and 213 ug mL^−1^ L‐ascorbic acid 2‐phosphate trisodium salt on day 11 for 4 days. Following purification, iPSC‐CMs were replated as monolayers (300000 cells cm^−2^) on growth factor reduced Matrigel (Corning) in RPMI 1640 media supplemented with B27 for 7 additional days before seeding into tissues.

### Microtissue Seeding and Culture

Due to the suspended nature of the fibrous matrices, it was determined that iPSC‐CMs must directly land on the top of the matrices by patterning the cells through a microfabricated cell seeding mask to prevent cells from “rolling” off the tops of the matrices and landing on the substrate beneath. Additionally, this technique significantly reduced the number of iPSC‐CMs needed to efficiently seed all the tissue on the substrate. To fabricate the seeding mask, 3D printed molds were designed in SolidWorks and printed via stereolithography (Protolabs). PDMS (1:10 crosslinker:base ratio) devices were replica cast from these molds. Prior to seeding the tissues, cell seeding masks were plasma treated for 5 min to generate highly hydrophilic surfaces that allow water to wick through the small microtissue scale wells in the mask. Vacuum grease was then applied to the edge of the seeding mask to ensure a watertight seal between the seeding mask and the substate prior to placing the mask on the tissue array substrate such that the holes in the mask sit directly above each suspended fiber matrix.

After aligning the seeding masks, iPSC‐CMs were dissociated by 0.25% Trypsin‐EDTA (Gibco) with 5% (v/v) Liberase for 5 min, stopped by an equal volume of 20% FBS/1 mM EDTA/PBS. Cells were triturated by gently pipetting with a p1000 pipette eight times to obtain a near single‐cell suspension and centrifuged (200 g, 4 min). iPSC‐CMs were resuspended in replating media (RPMI plus B27 supplement with 2% FBS and 5 µm Y‐27632 (Santa Cruz Biotechnology)) and 125000 cells were seeded per fibroTUG substrate through the top of the cell seeding mask in ≈200 µL of media. Cultures were then moved to the incubator and left undisturbed overnight to allow the iPSC‐CMs to attach to the tissues before removing the seeding masks. Cultures were maintained in RPMI media plus B27 supplement and replenished every other day for the duration of the studies. All studies were carried out for 7 days unless otherwise specified. To promote iPSC‐CM maturation in long‐term culture, tissues were cultured in OxPhos media composed of 25%glucose‐free RPMI and 75% glucose‐free DMEM with 1× B27 supplement and galactose, lactate, glutamax, and pyruvate at final media concentrations of 4, 4, 2, and 0.5 mM, respectively, as previously described.^[^ ^18^, ^73^ ^]^

### Contractile Force Analysis

Time‐lapse videos of the microtissue's spontaneous contractions were acquired at 65 Hz on a Zeiss LSM800 equipped with an Axiocam 503 camera while maintaining a temperature of 37 °C and 5% CO2. Maximum contractile force, contractile stress, contraction kinetics, and contraction frequency were calculated using a custom Matlab script based on the deflection of the posts and the measured post bending stiffness, as described previously.^[^ ^22^ ^]^ For the isoproterenol challenge, the same tissues were imaged prior to the addition of 10 nM isoproterenol and again 30 min after the addition of the drug for comparison.

### Calcium Imaging

Calcium handling analysis was performed by incubating cells for 1 h at 37 °C with 5 µm Cal520‐AM (AAT Bioquest). Cells were then returned to conditioned media preserved prior to adding the calcium sensitive dye and allowed to equilibrate for >30 min at 37 °C and 5% CO_2_. Following equilibration, tissues were imaged under epifluorescence at 65 Hz while maintaining temperature and CO_2_. Time‐lapse movies of calcium flux were analyzed with custom Matlab scripts as previously described.^[^ ^15^ ^]^ Briefly, average fluorescent profiles over time were determined for each tissue, and parameters such as beats per minute, peak‐to‐peak irregularity, flux rise time, flux decay time, and peak full width half max were calculated. The calcium transient correlation coefficient was determined by dividing the entire tissue into 16 regions of equal area and calculating the average Pearson's correlation coefficient between the flux profiles of each of these regions.

### Immunofluorescence Staining

Samples were fixed in 2% paraformaldehyde for 10 min at RT. Samples were then permeabilized in PBS solution containing Triton X‐100 (0.2% v/v), sucrose (10% w/v), and magnesium chloride (0.6% w/v) for 10 min and blocked in 1% (w/v) bovine serum albumin. Alternatively, to extract cytoplasmic vinculin, samples were simultaneously fixed and permeabilized in 2% paraformaldehyde in a buffer containing 1,4‐piperazinediethanesulfonic acid (PIPES, 0.1 m), ethylene glycol‐bis(2‐aminoethylether)‐N,N,N’,N’‐tetraacetic acid (EGTA, 1 mM), magnesium sulfate (1 mM), poly(ethylene glycol) (4% w/v), and triton X‐100 (1% v/v) for 10 min at room temperature, prior to blocking in 1% (w/v) bovine serum albumin. Tissues were incubated with rabbit monoclonal anti‐N‐cadherin (1:500; Abcam Ab18203), rabbit monoclonal anti‐connexin43 (1:1000; Millipore Sigma AB1728), mouse monoclonal anti‐α‐actinin (1:500; Abcam ab9465), mouse monoclonal anti‐cardiac troponin T (1:500; ThermoFisher MA5‐12960), mouse monoclonal anti‐vinculin (1:1000; Millipore Sigma V9264), rabbit polyclonal anti‐myosin light chain 2 (1:500; Proteintech 10906‐1‐AP), rabbit polyclonal anti‐non‐muscle myosin II‐B (1:1000; Biolegend 909902), mouse monoclonal anti‐integrin β1D (1:1000; Abcam ab8991), rabbit polyclonal anti‐zyxin (1:200; Millipore Sigma HPA004835), or mouse anti‐dextran (1:500; STEMCELL Technologies 60026) antibodies for 1 h at RT, followed by goat anti‐mouse Alexa Fluor 647 (1:1000; Life Technologies A21236), goat anti‐mouse Alex Fluor 546 (1:1000, Life Technologies A11030), or goat anti‐rabbit Alexa Fluor 647 secondary antibodies (1:1000; Life Technologies A21245) and DAPI for 1 h at RT.

### Microscopy and Image Analysis

Fluorescent images were captured on a Zeiss LSM800 confocal microscope. Sarcomere alignment was quantified via custom Matlab scripts as previously described.^[^ ^15^ ^]^ Briefly, images of the titin‐GFP reporter were thresholded, and individual z‐discs were segmented. Z‐discs were subsequently grouped with neighboring z‐discs based on proximity and orientation to identify myofibrils within the image. The orientation of all identified myofibrils within a field of view was fit to a Gaussian distribution. Sarcomere alignment deviation was defined at the standard deviation of this distribution using circular/angular statistics. Myofibril density was calculated by determining the percent area of each tissue containing titin‐rich myofibril structures.

Vinculin and N‐cadherin morphology and colocalization analysis were also performed using custom Matlab scripts. Because of the 3D nature of these tissues due to the intercalation of iPSC‐CMs into matrix pores, confocal z‐stacks of fibroTUG tissues formed with iPSC‐CMs containing a GFP‐titin live‐reporter and immunostained for vinculin and N‐cadherin were segmented in 3D for vinculin‐, N‐cadherin‐, and titin‐enriched structures. Custom Matlab functions were implemented to extract volume, eccentricity, and other parameters for these structures. Where applicable, quantifications were normalized to cell number in each field of view.

### Computational Modeling

The development of a tissue specific finite element model of fibroTUG tissues was described in detail by Jilberto et al. in an accompanying manuscript.^[^ ^67^ ^]^ Briefly, the images of the DVS fibers and titin were processed using Matlab/Python scripts to quantify the specific fiber structure and a probabilistic characterization of the myofibril organization.^[^ ^103^ ^]^ This information was projected into a 2D triangular finite element mesh. Using these quantities, and following a continuum mechanics approach, non‐linear constitutive relationships for the fibers and cells were defined. The mechanical response of the tissue was taken to be the sum of these two components. Methods similar to those presented in Miller et al.^[^ ^104^ ^]^ were then adapted to find the necessary active stress that the cells were exerting to generate the observed boundary tractions and displacement conditions. Using the experimental data for each of the permutations of interest (soft/stiff fibers with soft/stiff post), more than a hundred *in‐silico* tissues were generated for each of them by combining image‐derived fibrous matrix with probabilistic‐generated myofibril fields and experimentally measured force responses specific for each condition. The results were compiled to obtain a distribution of active stress for each mechanical environment.

### Statistical Analysis

Statistical significance was determined by t‐tests and one‐way or two‐way analysis of variance (ANOVA) with post‐hoc analysis (Tukey test), where appropriate, with significance indicated by *p* < 0.05. Studies were conducted a minimum of 3 times in each experiment. The data presented were representative data sets from one of these replicate studies. Specific sample size was indicated within corresponding figure legends and all data were presented as mean ± standard deviation.

## Conflict of Interest

The authors declare no conflict of interest.

## Author Contributions

Conceptualization was done by S.J.D. and B.M.B. Methodology was done by S.J.D., J.J., A.E.S., D.D.H., C.D.D., H.B., R.N.K., E.L., A.S.H., D.A.N., and B.M.B. Investigation was done by S.J.D., J.J., A.E.S., D.D.H., J.L., A.C., R.N.K., M.E.J., and H.K. Visualization was done by S.J.D. Supervision was done by C.S.C., E.L., A.S.H., D.A.N., and B.M.B. Writing—original draft was done by S.J.D. and B.M.B. Writing—review and editing was done by S.J.D., J.J., A.E.S., D.D.H., R.N.K., M.E.J., H.K., C.S.C., E.L., A.S.H., D.A.N., and B.M.B. Funding acquisition was done by S.J.D., C.S.C., E.L., A.S.H., D.A.N., and B.M.B.

## Supporting information

Supporting Information

Supplemental Video 1

Supplemental Video 2

Supplemental Video 3

Supplemental Video 4

Supplemental Video 5

Supplemental Video 6

Supplemental Video 7

Supplemental Video 8

Supplemental Video 9

Supplemental Video 10

## Data Availability

The data that support the findings of this study are available from the corresponding author upon reasonable request.
