# Supplementary material for: Matrix Architecture and Mechanics Regulate Myofibril Organization, Costamere Assembly, and Contractility in Engineered Myocardial Microtissues
Source: Adv Sci (Weinh). 2024 Nov 18;11(47):2309740. doi: 10.1002/advs.202309740 (PMC11653763; doi:10.1002/advs.202309740)
Supplement: Supplementary file 1 — Supporting Information [file ADVS-11-2309740-s011.docx]

## Supplemental Information

**Matrix Architecture and Mechanics Regulate Myofibril Organization, Costamere Assembly, and Contractility in Engineered Myocardial Microtissues**

Samuel J. DePalma^1^, Javiera Jillberto^1^, Austin E. Stis^1^, Darcy D. Huang^1^, Jason Lo^1^, Christopher D. Davidson^1^, Aamilah Chowdhury^1^, Robert N. Kent III^1^, Maggie E. Jewett^1^, Hiba Kobeissi^2^, Christopher S. Chen^6,7^, Emma Lejeune^2^, Adam S. Helms^3^, David A. Nordsletten^1,4,5^, Brendon M. Baker^1,8,+^

^1^ Department of Biomedical Engineering, University of Michigan, Ann Arbor, MI 48109

^2^ Department of Mechanical Engineering, Boston University, Boston, MA 02215

^3^ Division of Cardiovascular Medicine, University of Michigan, Ann Arbor, MI 48109

^4^ Department of Cardiac Surgery, University of Michigan, Ann Arbor, MI 48109

^5^ Department of Biomedical Engineering, School of Imaging Sciences and Biomedical Engineering, King's College London, King's Health Partners, London SE1 7EH, United Kingdom

^6^ Department of Biomedical Engineering, Boston University, Boston, MA 02215, USA

^7^ Wyss Institute for Biologically Inspired Engineering, Harvard University, Boston, MA 02115, USA

^8^ Department of Chemical Engineering, University of Michigan, Ann Arbor, MI 48109

^+^ Corresponding Author:

Brendon M. Baker, Ph.D.

Assistant Professor, Department of Biomedical Engineering, University of Michigan

2174 Lurie BME Building, 1101 Beal Avenue

Ann Arbor, MI 48109

Email: bambren@umich.edu

## SUPPLEMENTAL FIGURES


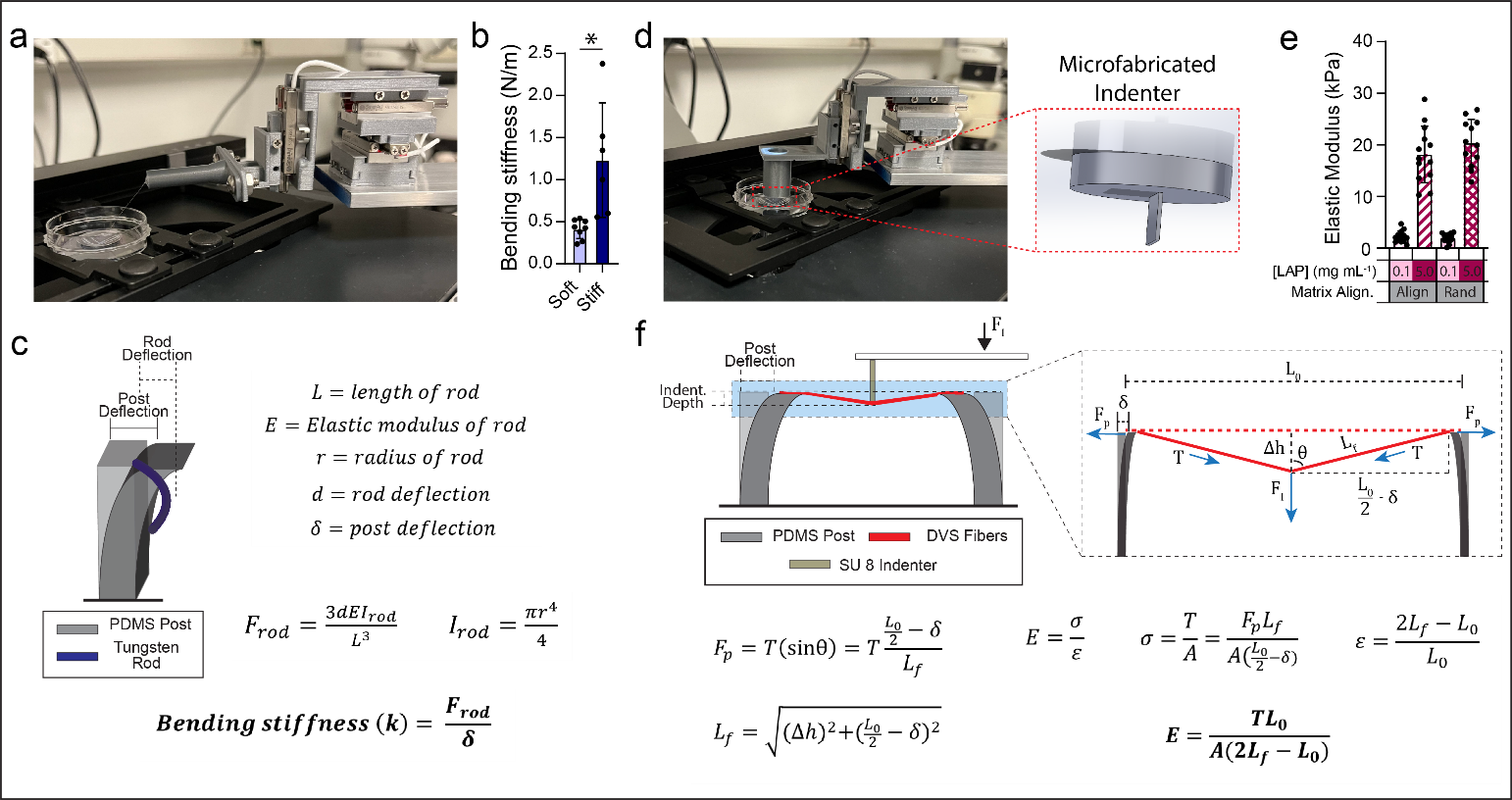


**Figure S1: Mechanical characterization of fibroTUG platform.** (**a**) Image of PDMS post mechanical characterization setup. (**b**) Measured bending stiffness of soft and stiff PDMS posts. (**c**) Schematic of post mechanical testing scheme and equations used to calculate post bending stiffness. (**d**) Image of fiber matrix mechanical characterization setup. (**e**) Measured elastic modulus of various fiber matrices. (**f**) Schematic of fiber matrix mechanical testing scheme and equations used to calculate matrix elastic modulus. All data presented as mean ± std; * p < 0.05.

**
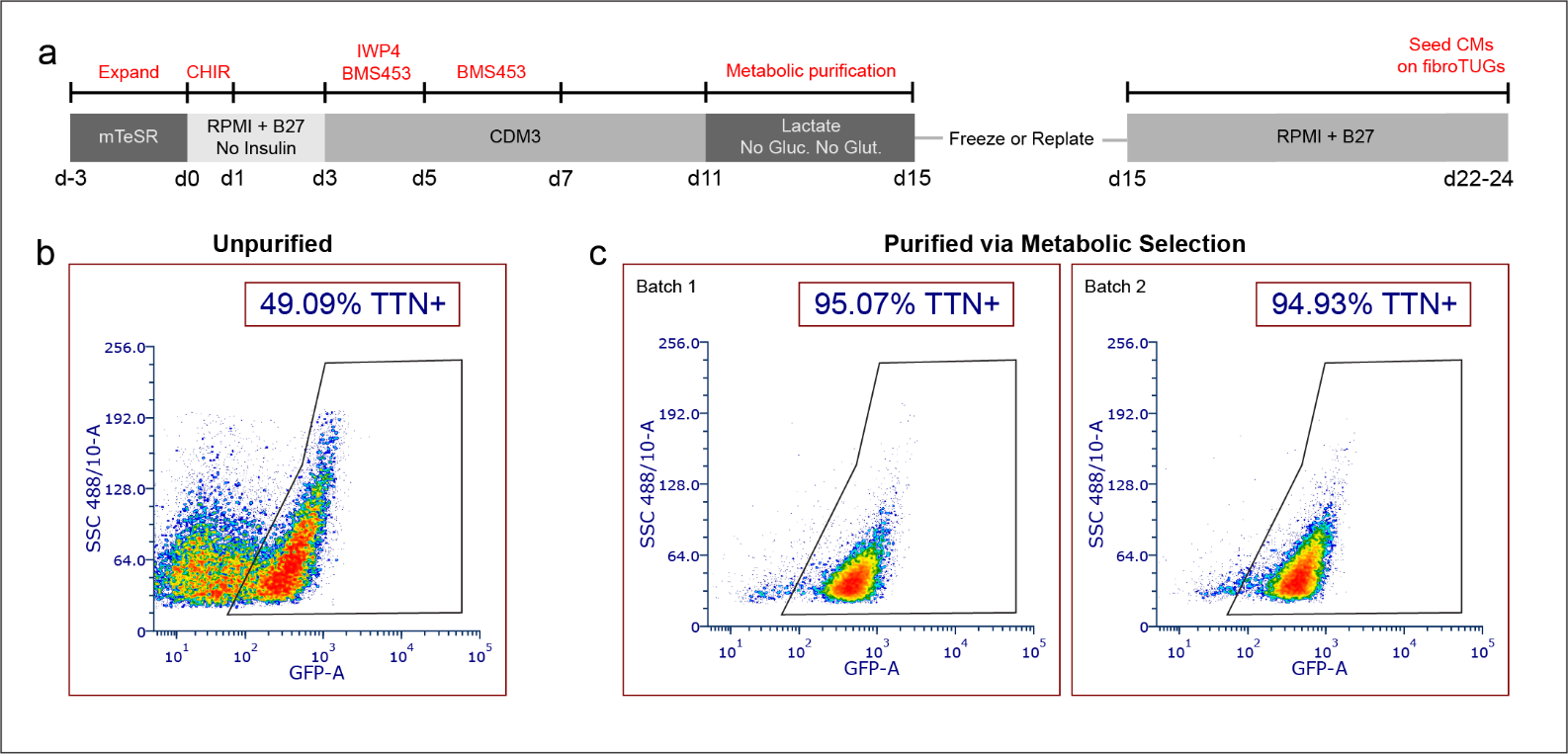
**

**Figure S2: Differentiation and purification of iPSC-CM cultures.** (**a**) Timeline illustrating differentiation of iPSC to CMs, metabolic purification of iPSC-CMs populations, and seeding of CMs on fibroTUG tissues. (**b**) Flow cytometry analysis of unpurified iPSC-CM populations measured at day 22 in the differentiation protocol showed that ~49% of cells expressed the CM marker TTN. (**c**) Flow cytometry analysis of metabolically selected iPSC-CM populations measured at day 22 in the differentiation protocol showed that ~95% of cells expressed the CM marker TTN. Analysis is shown for two separate differentiations to illustrate the consistency of this purification method.

**
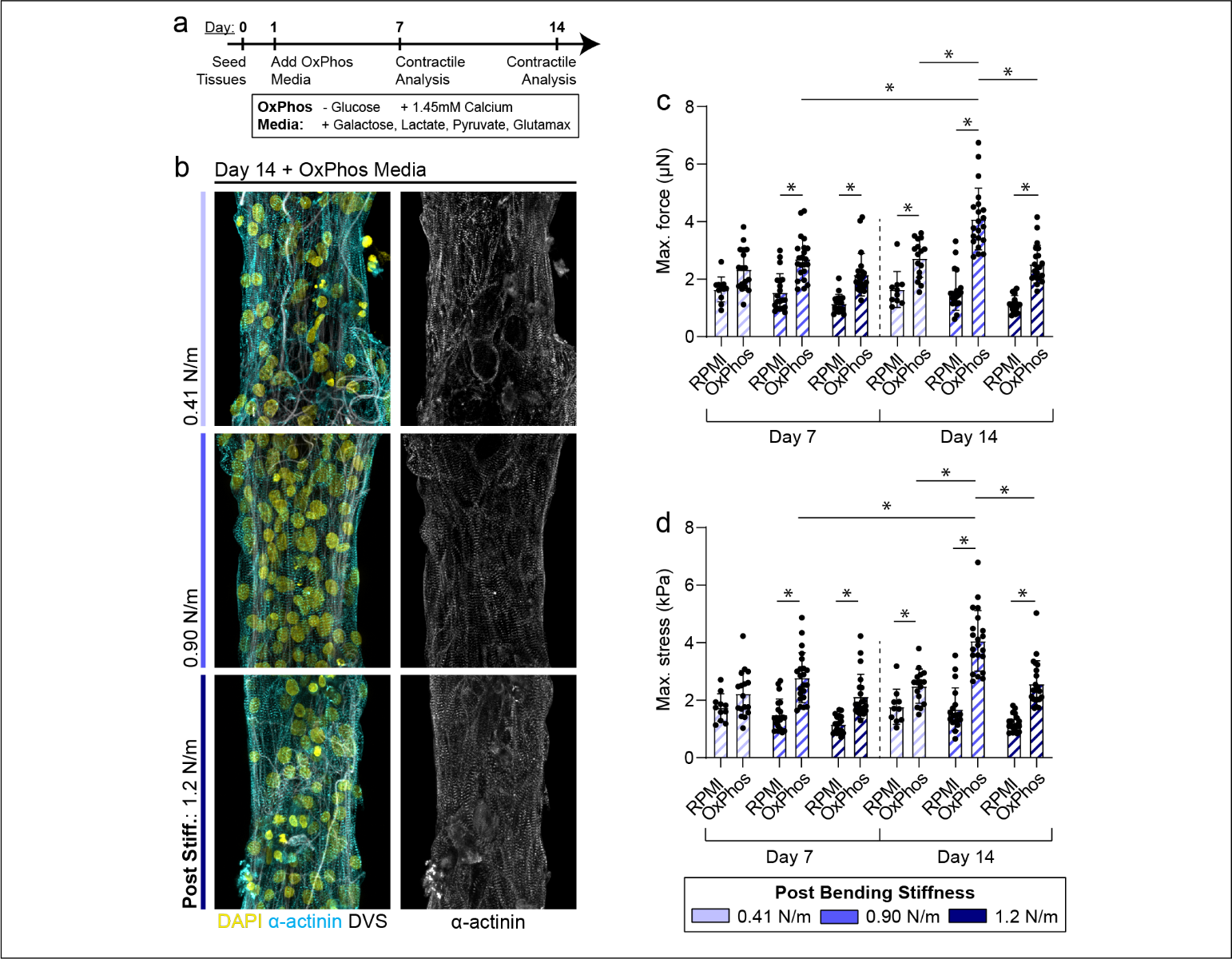
**

**Figure S3: fibroTUG platform supports long-term culture of iPSC-CMs with metabolic maturation media.** (**a**) Experimental timeline. (**b**) Confocal fluorescent images of fibroTUG tissues after 14 days in culture with OxPhos metabolic maturation media. (**c**) Max contractile force and (**d**) max contractile stress of tissues between various post stiffnesses in both RPMI B27 and OxPhos media. All data presented as mean ± std; * p < 0.05.

**
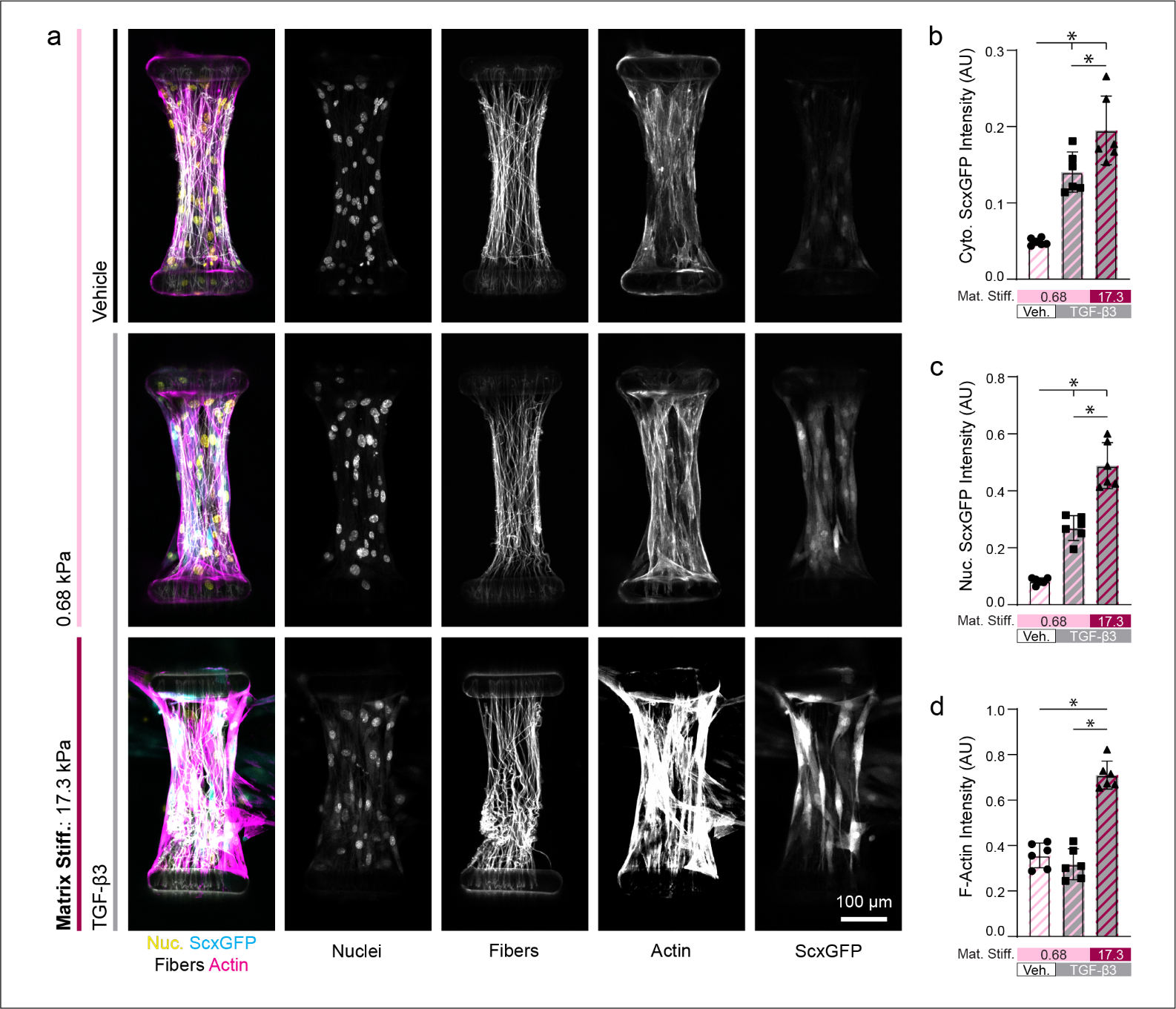
**

**Figure S4: Matrix stiffness drive tenogenic differentiation of tendon progenitor cells (TPCs) in fibroTUG platform.** (**a**) Confocal fluorescent images of fibroTUG tissues with stiff (1.2 N/m) posts, due to the high baseline contractility of TCPs, and aligned fibers of varying stiffness. TCPs were isolated from mice engineered to express a live GFP-scleraxis (Scx) report and were stained for actin using phalloidin. Tissues were also treated with T3 to induce further tenogenic differentiation. Quantification of cytosolic scleraxis expression (**b**), nuclear scleraxis expression (**c**), and actin expression (**d**) (n ≥ 6). All data presented as mean ± std; * p < 0.05.

**
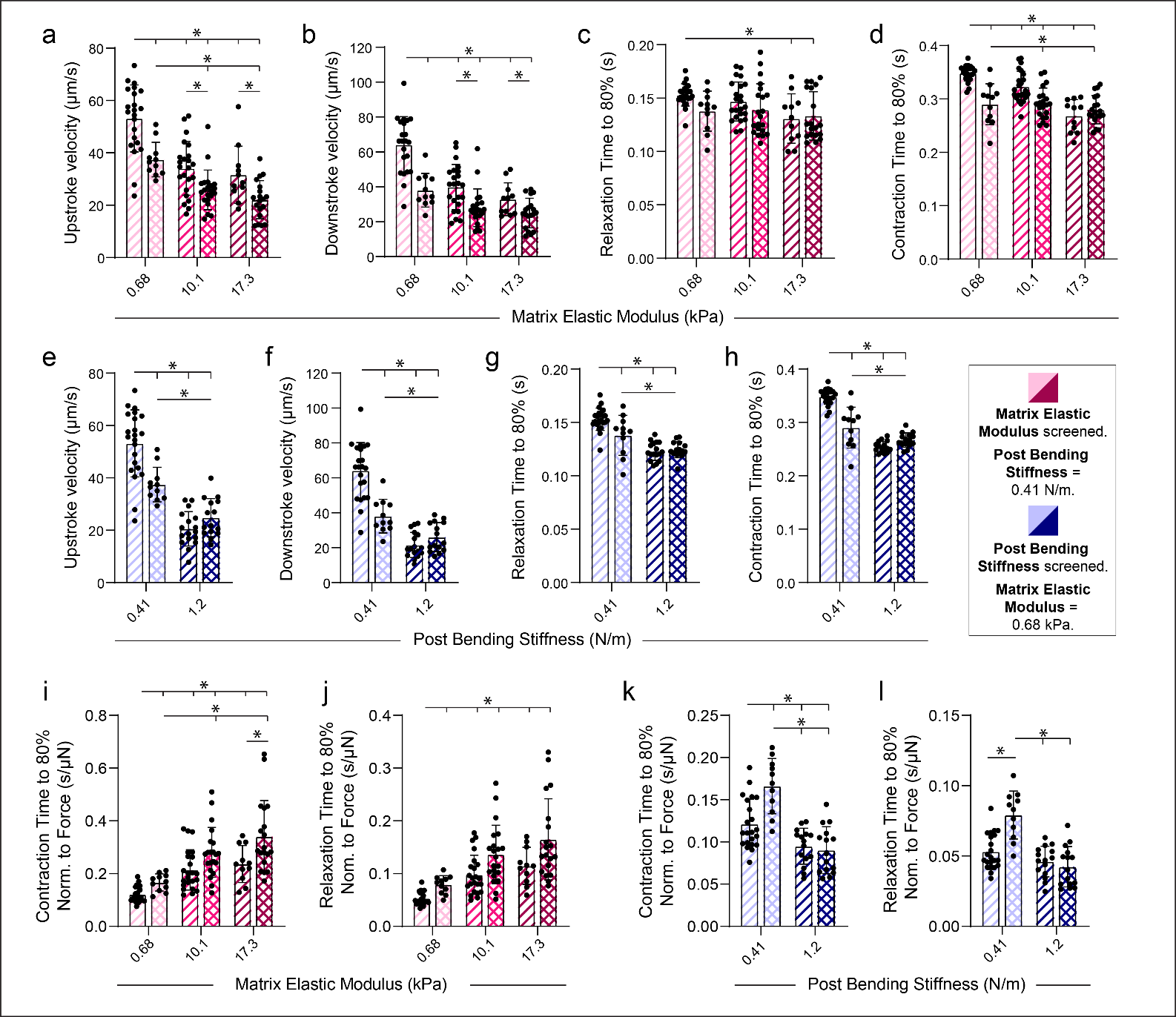
**

**Figure S5: Fibrous matrix alignment and stiffness influences iPSC-CM tissue contractile dynamics.** Upstroke velocity (**a**), downstroke velocity (**b**), relaxation time to 80% relaxation (**c**), and contraction time to 80% relaxation (**d**) quantified in tissues with constant post stiffness (0.41 N/m) with varied fiber alignment and fiber stiffness (n ≥ 11 tissues). Upstroke velocity (**e**), downstroke velocity (**f**), relaxation time to 80% relaxation (**g**), and contraction time to 80% relaxation (**h**) quantified in tissues with constant matrix stiffness (0.68 kPa) with varied fiber alignment and post stiffness (n ≥ 11). (**i-l**) Relaxation time to 80% and contraction time to 80% relaxation were also normalized to the contractile force (i.e. peak amplitude). All data presented as mean ± std; * p < 0.05.

**
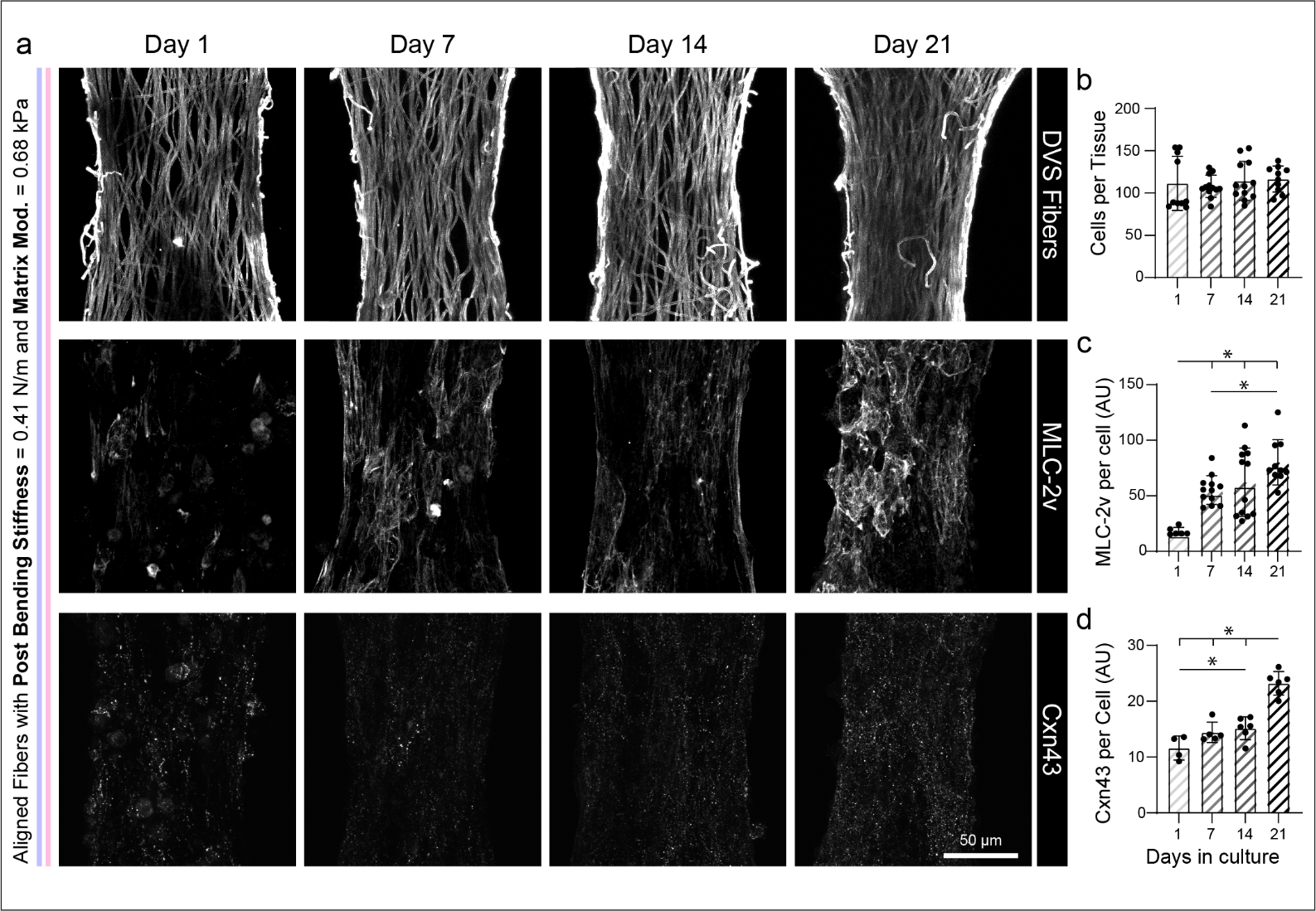
**

**Figure S6: Long-term culture of fibroTUG tissues in maturation medium leads to progressive structural maturation.** (**a**) Confocal fluorescent images of fibroTUG tissues with soft (0.68 kPa), aligned fibers and soft (0.41 N/m) post after 1, 7, 14, and 21 days in culture with OxPhos metabolic maturation medium immunostained for dextran (DVS fibers), MLC-2v, and connexin-43. (**b**) Quantification of the number of cells per tissue over time (n ≥ 12). (**c**) Quantification of MLC-2v expression (n ≥ 12). (**d**) Quantification of connexin-43 expression (n ≥ 6). All data presented as mean ± std; * p < 0.05.

**
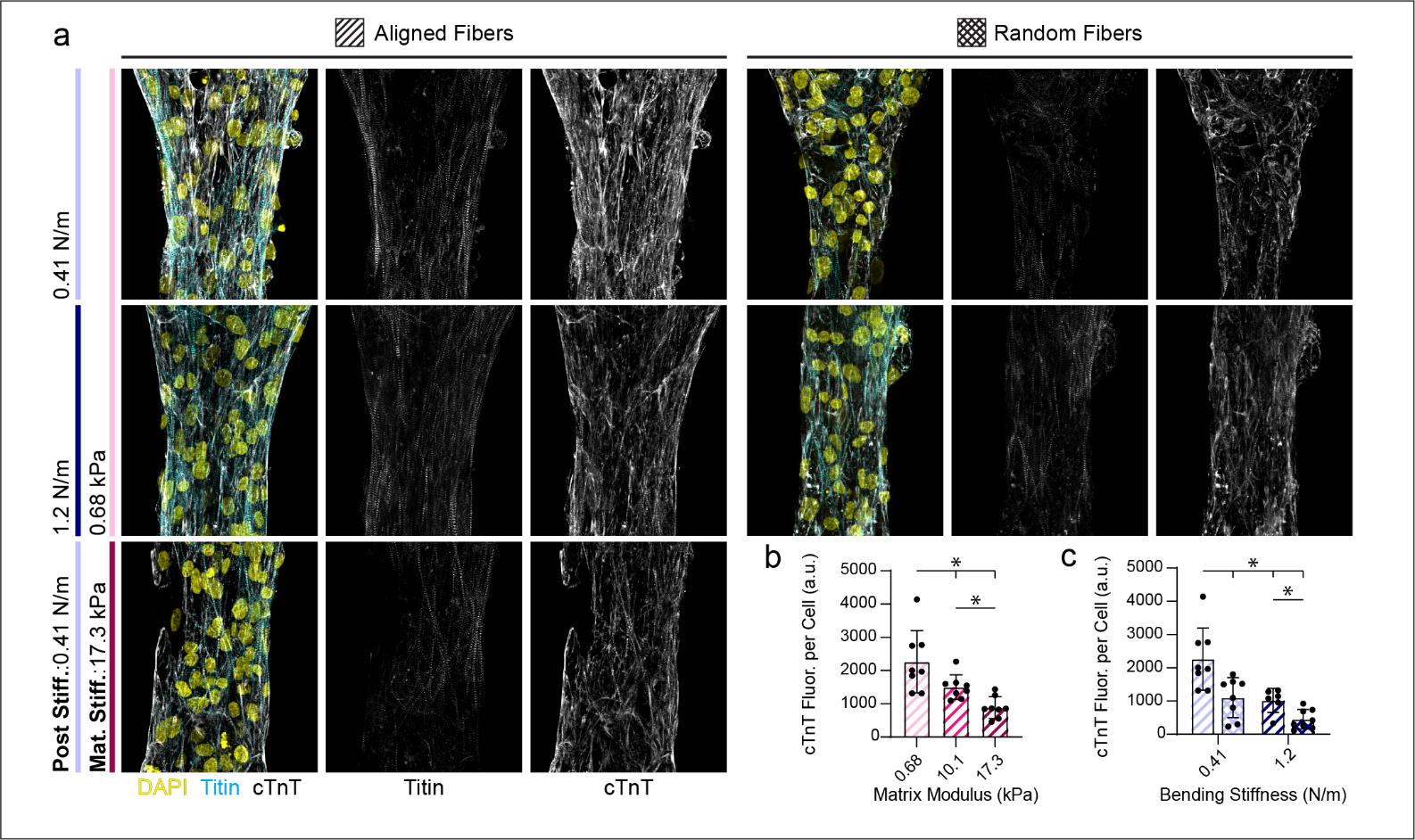
**

**Figure S7: Cardiac troponin expression in fibroTUG tissues.** (**a**) Confocal fluorescent images of fibroTUG tissues of varying mechanics immunostained for cTnT. Quantification of cTnT fluorescence per cell in tissues with (**b**) varied matrix stiffness and (**c**) bending stiffness. All data presented as mean ± std; * p < 0.05.

**
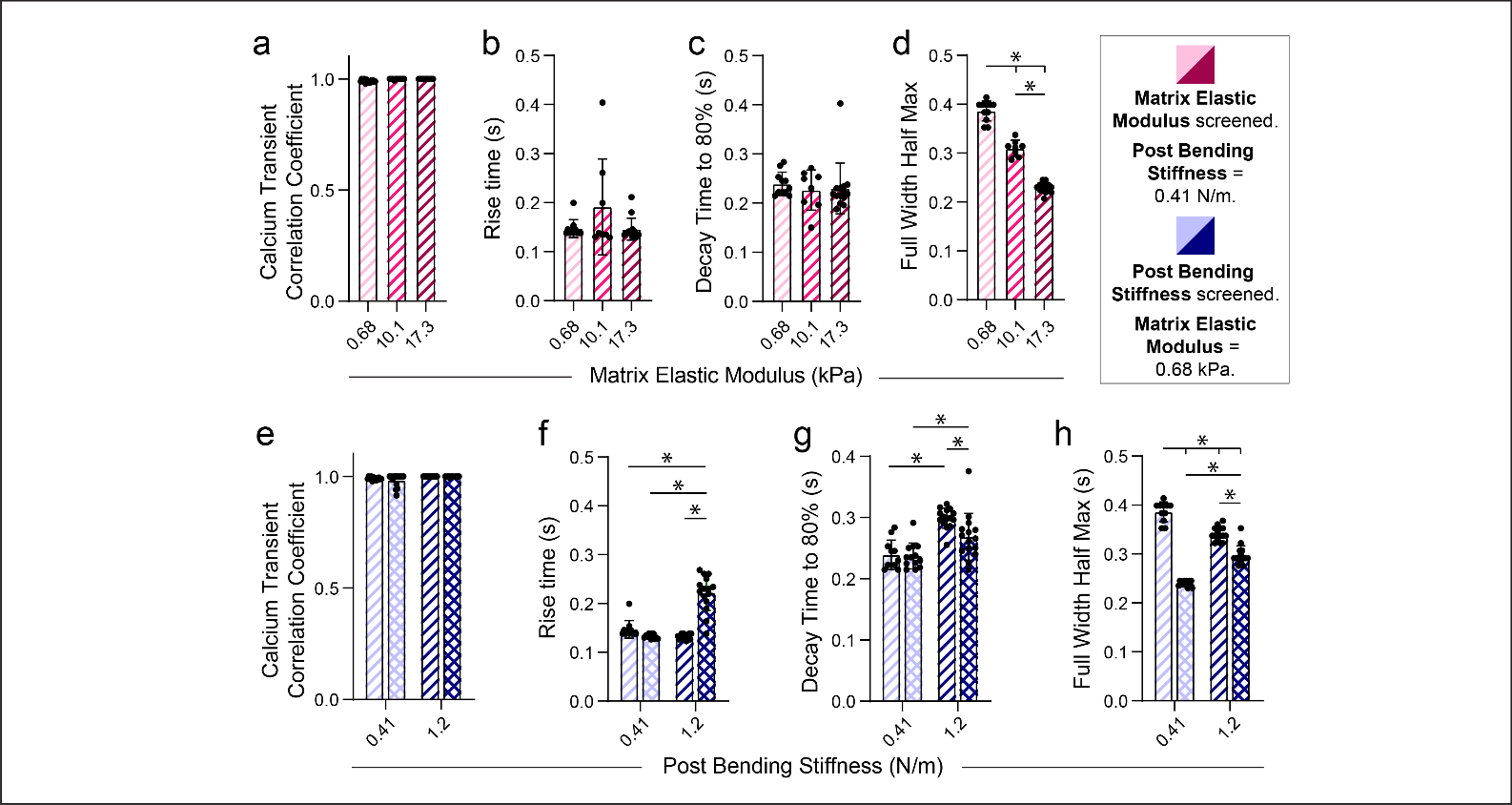
**

**Figure S8: Fibrous matrix alignment and stiffness influences iPSC-CM tissue calcium flux dynamics.** Calcium transient correlation coefficient (**a**), calcium flux rise time (**b**), decay time to 80% relaxation (**c**), full width half max (**d**) quantified in tissues with aligned fibers and constant post stiffness (0.41 N/m) with varied fiber stiffness (n ≥ 11). Contraction correlation coefficient (**e**), calcium flux rise time (**f**), decay time to 80% relaxation (**g**), full width half max (**h**) quantified in tissues with constant fiber stiffness (0.68 kPa) with varied post stiffness and fiber alignment (n ≥ 11). All data presented as mean ± std; * p < 0.05.


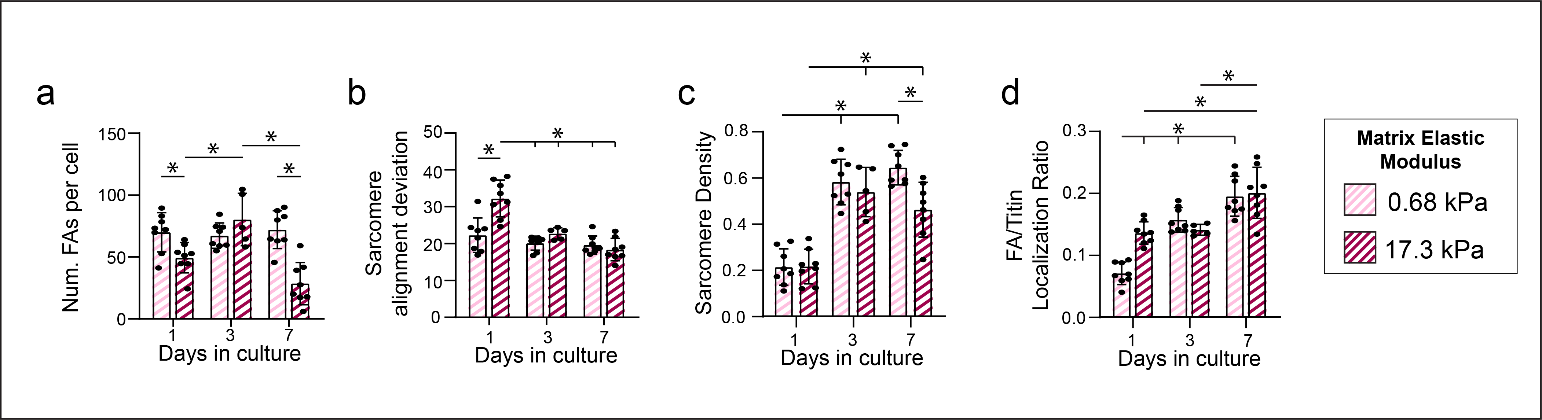


**Figure S9: Matrix mechanics influence costamere formation and sarcomere formation over time.** Quantification of number of (**a**) focal adhesions per cell, (**b**) sarcomere alignment, (**c**) sarcomere density, (**d**) and the fraction of vinculin that is colocalized with titin in tissues formed on aligned soft and stiff matrices suspended between 0.41 N/m posts fixed at 1-, 3-, and 7-days post seeding (n ≥ 5). All data presented as mean ± std; * p < 0.05.


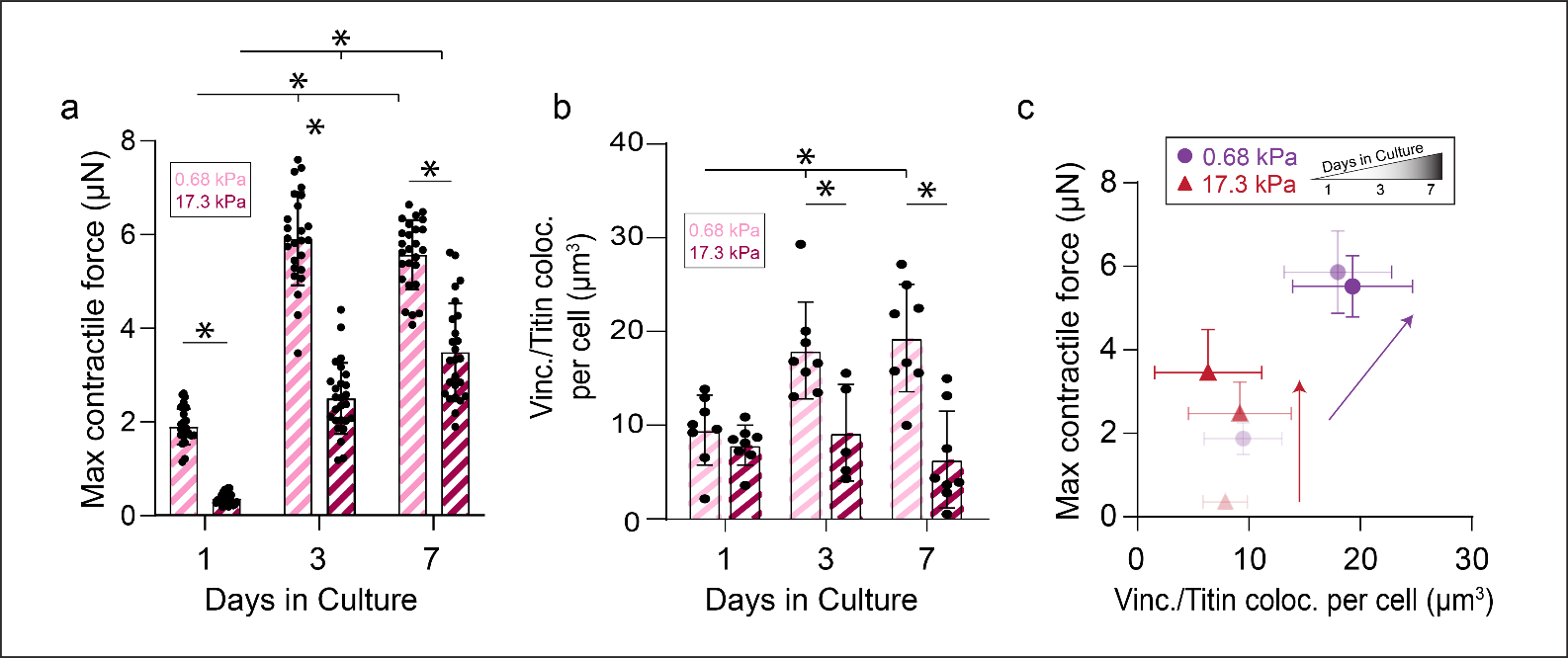


**Figure S10: Increased tissue contractile function is associated with costamere formation on soft matrices.**

(**a**) Quantification of max contractile force of fibroTUG tissues at day 1, 3 and 7 post seeding on either soft (0.68 kPa) or stiff (17.1 kPa) aligned fiber matrices (post stiffness was held constant at 0.41 N/m) (n ≥ 20). (**b**) Costamere formation was assessed by quantifying vinculin colocalization with titin from confocal fluorescent images of fibroTUG tissues fixed at day 1, 3 and 7 post seeding on either soft (0.68 kPa) or stiff (17.1 kPa) aligned fiber matrices (post stiffness was held constant at 0.41 N/m) (n ≥ 5). (**c**) Plot showing average tissue contractile force at days 1, 3, and 7 vs. costamere formation (as quantified by vinculin colocalization with titin).


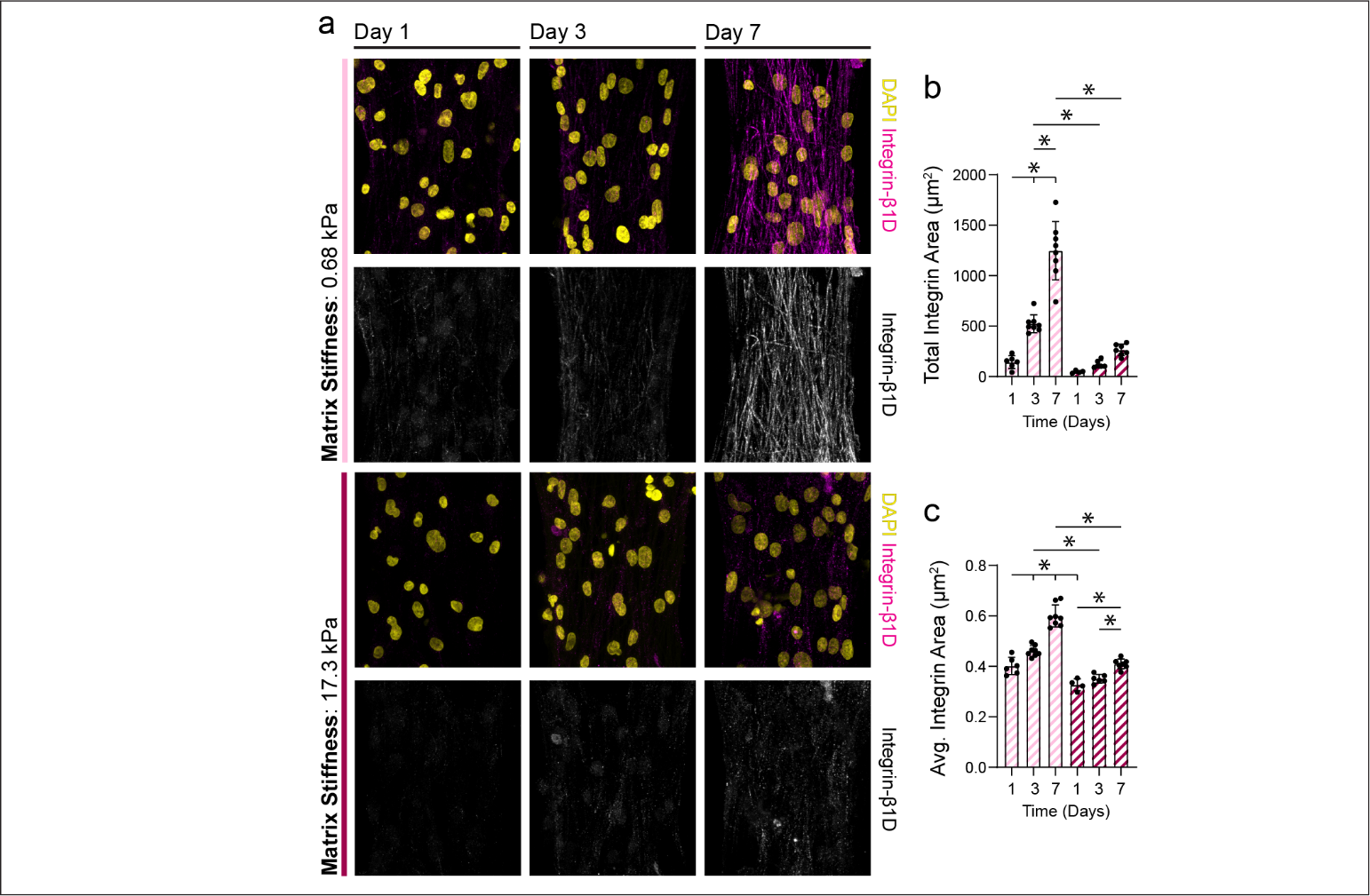


**Figure S11: Quantification of β1D integrin expression in fibroTUG tissues.** (**a**) Confocal fluorescent images of fibroTUG tissues fixed at day 1, 3 and 7 post seeding on either soft (0.68 kPa) or stiff (17.1 kPa) aligned fiber matrices (post stiffness was held constant at 0.41 N/m). Quantification of (**b**) total integrin area and (**c**) average integrin size (n ≥ 5). All data presented as mean ± std; * p < 0.05.


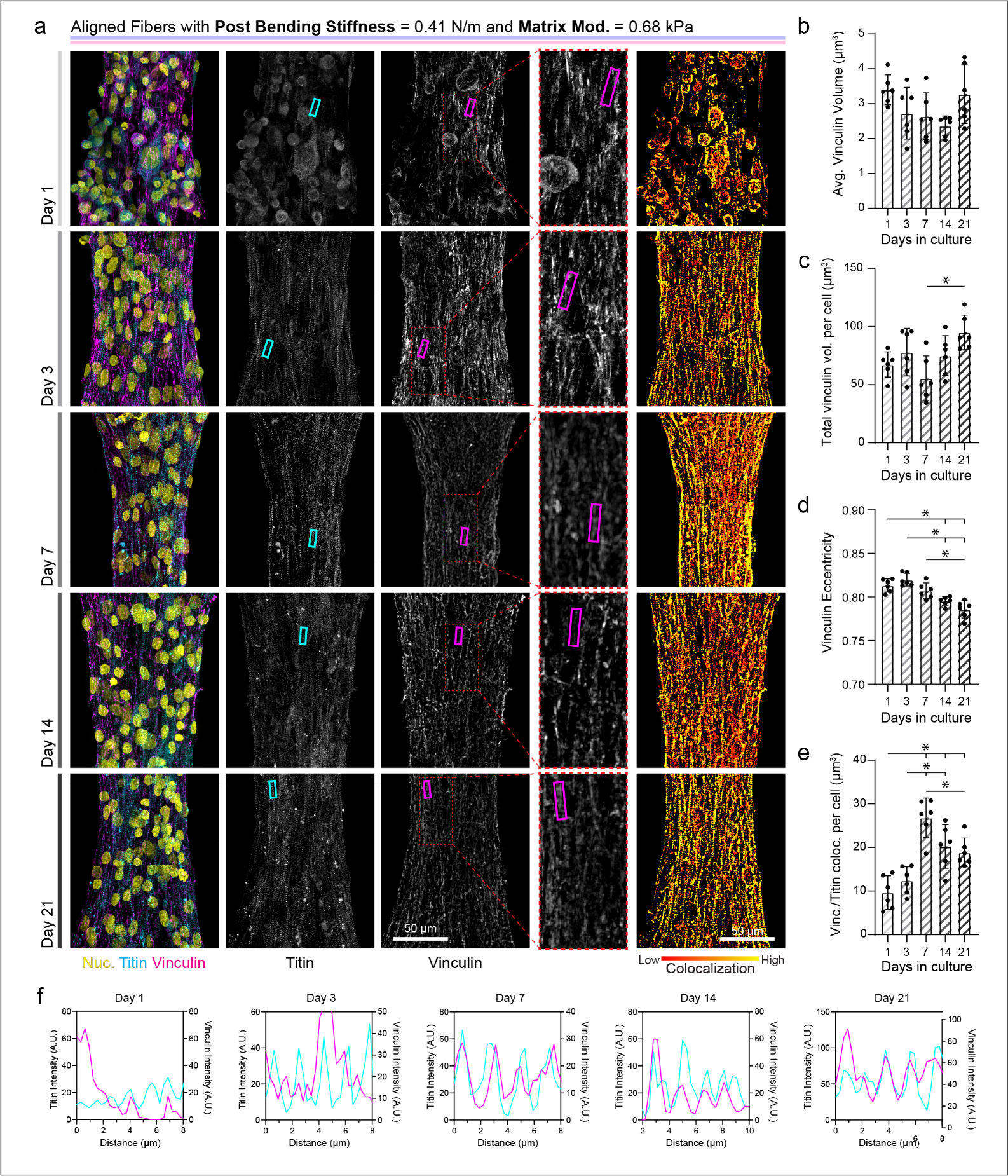


**Figure S12: Soft, aligned fibers and soft posts facilitate robust costamere formation in long term culture.** (**a**) Confocal fluorescent images of fibroTUG tissues reporter fixed at day 1, 3, 7, 14, and 21 post seeding on aligned, soft (0.68 kPa) matrices between soft (0.41 N/m) posts containing a GFP-titin and immunostained for vinculin. Tissues were cultured in OxPhos maturation medium. (**b**) Average vinculin volume, (**c**) total vinculin volume, (**d**) and vinculin eccentricity were quantified from the fluorescent images of immunostained vinculin (n ≥ 6). (**e**) Costamere formation was assessed by quantifying vinculin colocalization with titin (n ≥ 6). (**f**) Colocalization of vinculin and titin was visualized via fluorescence intensity plots of titin (cyan) and vinculin (magenta) at all time points from lines drawn along the major axis of regions indicated by the rectangles overlayed on images in panel a. All data presented as mean ± std; * p < 0.05. All data presented as mean ± std; * p < 0.05.


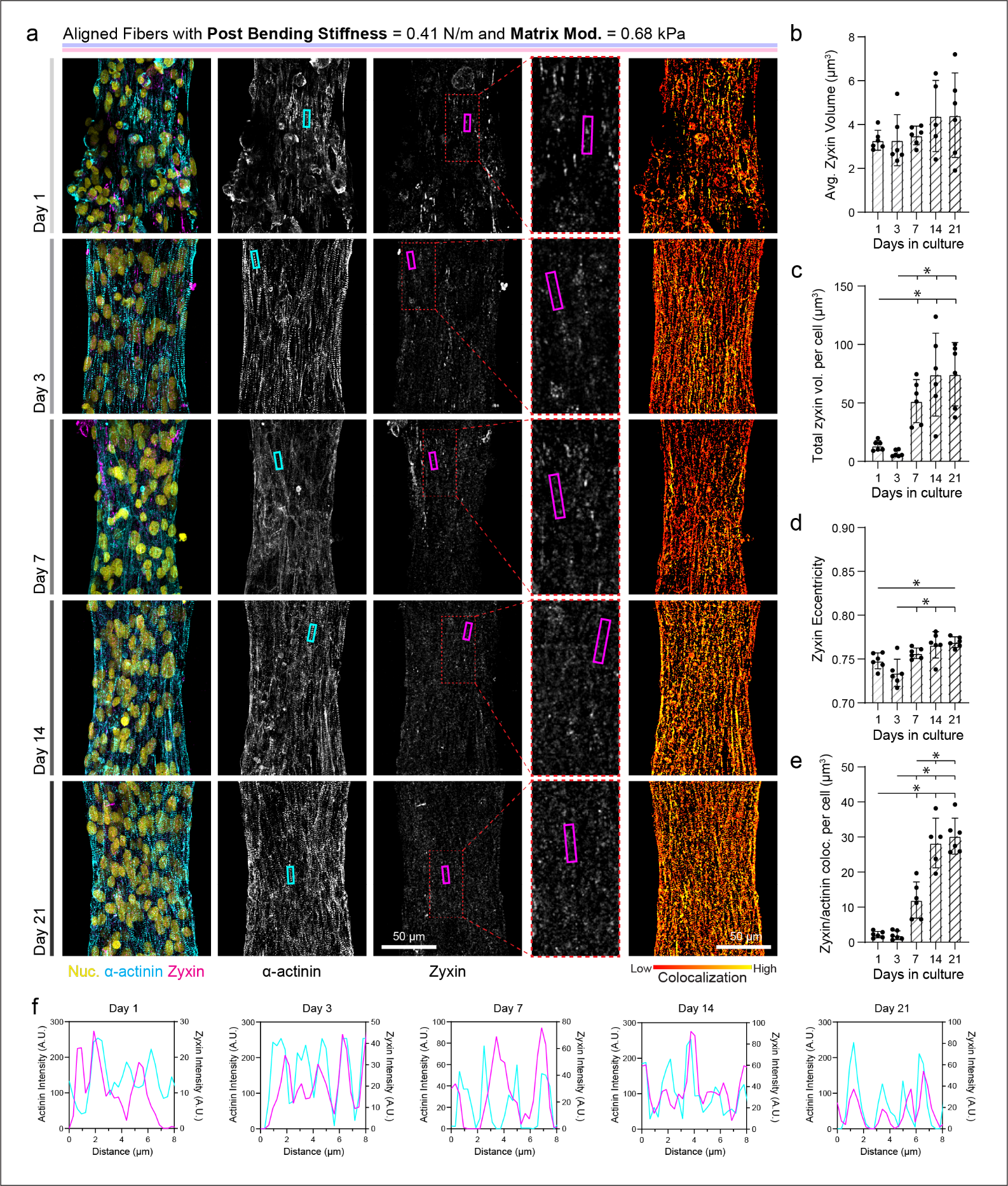


**Figure S13: Soft, aligned fibers and soft posts facilitate robust zyxin localization to costameres in long term culture.** (**a**) Confocal fluorescent images of fibroTUG tissues fixed at day 1, 3, 7, 14, and 21 post seeding on aligned, soft (0.68 kPa) matrices between soft (0.41 N/m) posts immunostained for α-actinin and zyxin. Tissues were cultured in OxPhos maturation medium. (**b**) Average zyxin volume, (**c**) total zyxin volume, (**d**) and zyxin eccentricity were quantified from the fluorescent images of immunostained zyxin (n ≥ 6). (**e**) Costamere formation was assessed by quantifying zyxin colocalization with α-actinin (n ≥ 6). (**f**) Colocalization of zyxin and α-actinin was visualized via fluorescence intensity plots of α-actinin (cyan) and zyixin (magenta) at all time points from lines drawn along the major axis of regions indicated by the rectangles overlayed on images in panel a. All data presented as mean ± std; * p < 0.05. All data presented as mean ± std; * p < 0.05.

**
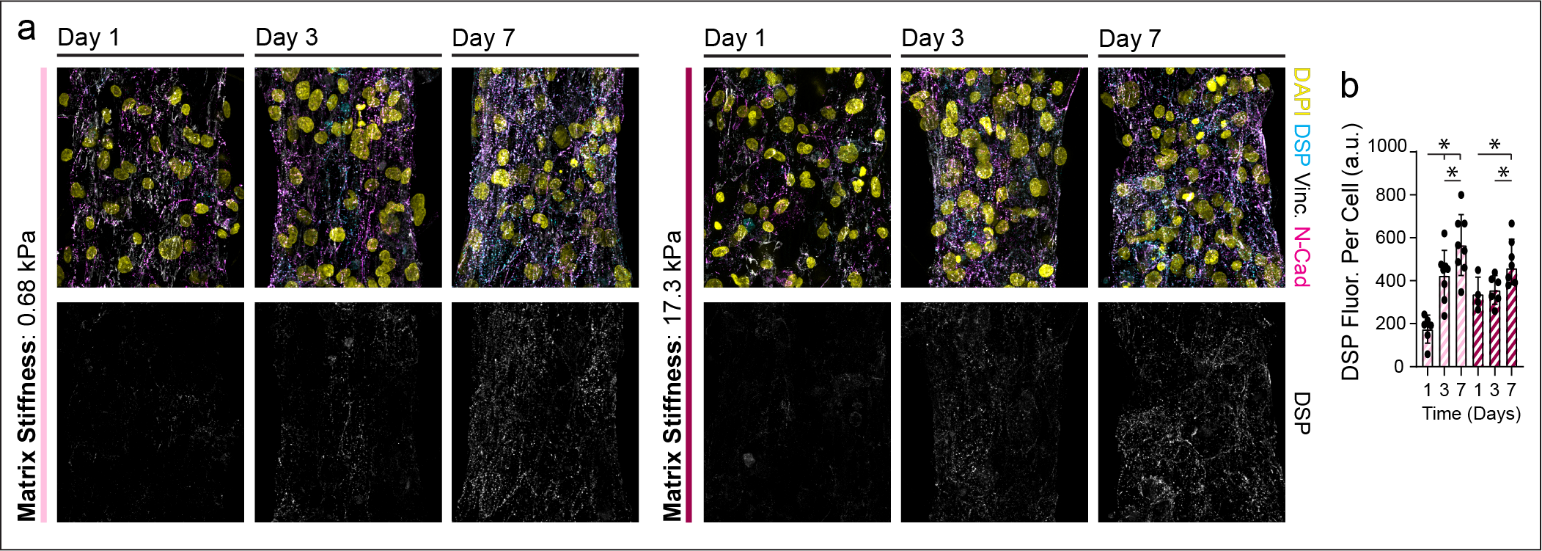
**

**Figure S14: Intercalated disc formation over time on soft and stiff fiber matrices.** (**a**) Confocal fluorescent images of fibroTUG tissues fixed at day 1, 3 and 7 post seeding on either soft (0.68 kPa) or stiff (17.1 kPa) aligned fiber matrices (post stiffness was held constant at 0.41 N/m). (**b**) Quantification of DSP fluorescence per cell (n ≥ 8). All data presented as mean ± std; * p < 0.05.


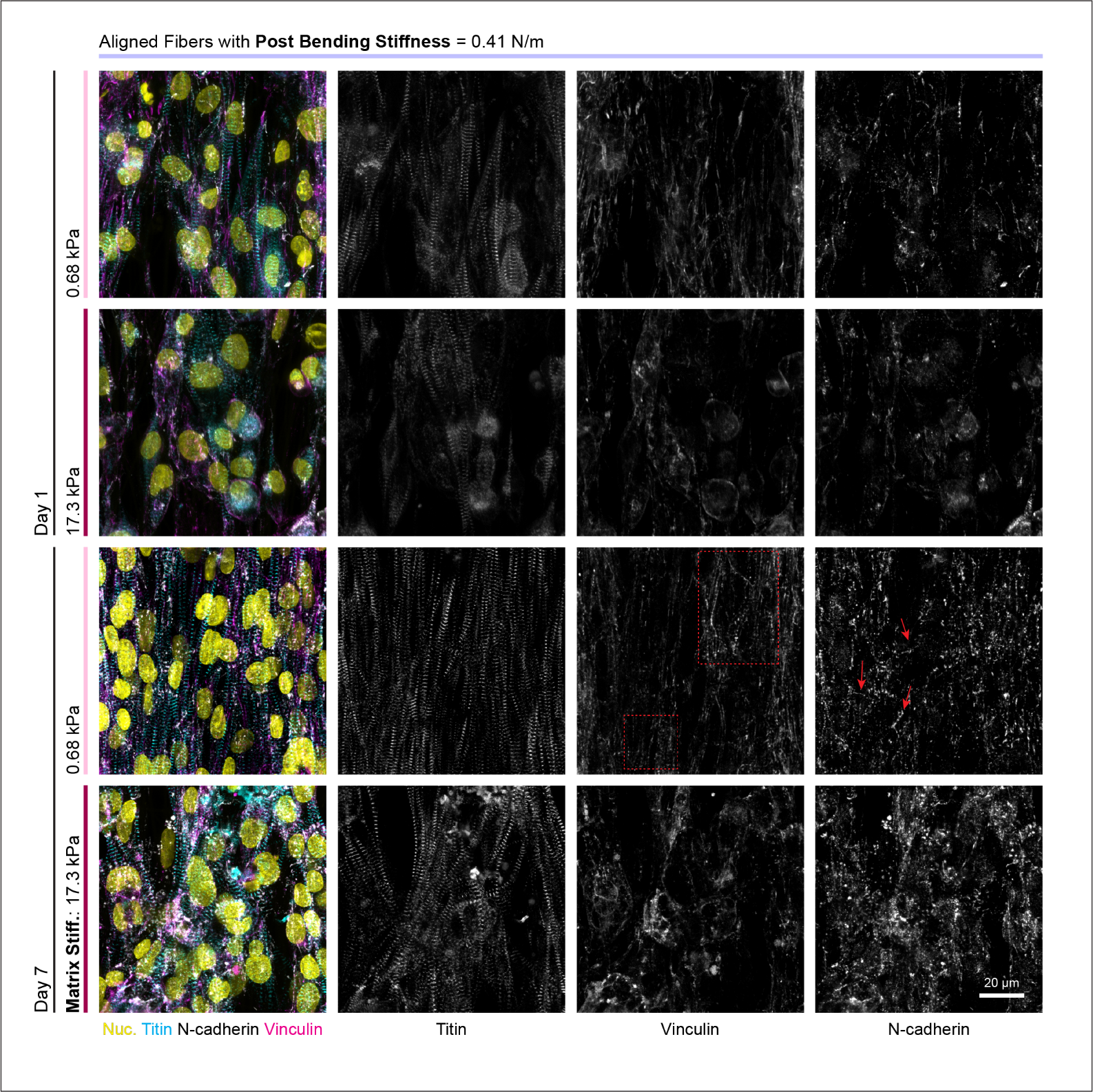


**Figure S15: High magnification images of fibroTUG tissues show robust costamere and intercalated disc formation on soft, aligned fiber matrices.** (**a**) Confocal fluorescent images of fibroTUG tissues fixed at day 1 and 7 after seeding on either soft (0.68 kPa) or stiff (17.1 kPa) aligned fiber matrices (post stiffness was held constant at 0.41 N/m) acquired at 63x magnification. All images show a region located at the center of each tissue.


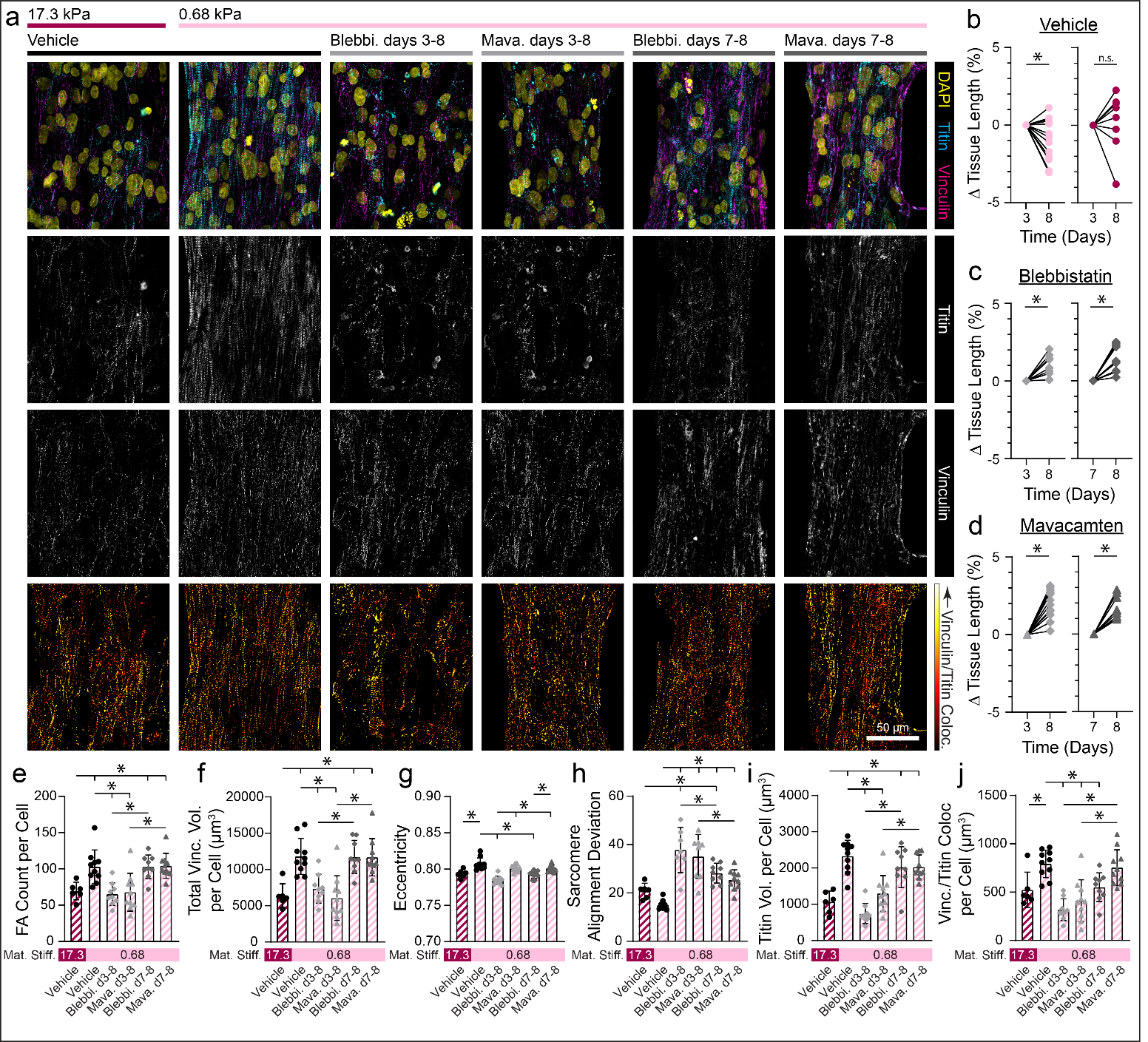


**Figure S16: Treatment with contractile inhibitors at day 3 and day 7.** (**a**) Confocal fluorescent images of fibroTUG tissues treated with blebbistatin (50uM) or mavacamten (500 nM) starting at either day 3 or day 7post seeding. (**b**) Diastolic tissue length on day 3 and 8 of tissues seeded on soft (0.68 kPa) and stiff (17.1 kPa) aligned fiber matrices (post stiffness was held constant at 0.41 N/m) without treatment with the contractile inhibitors. (**c,d**) Diastolic tissue length of tissues seeded on soft matrices on day 3 or day 7 before treatment with a contractile inhibitor, (**c**) blebbistatin or (**d**) mavacamten, and day 8 after 5 or 1 days of treatment,(n ≥ 8). (**e**) Focal adhesion count, (**f**) vinculin volume per cell, and (**g**) focal adhesion eccentricity were quantified from the fluorescent images of immunostained vinculin (n ≥ 6). (**h**) Sarcomere alignment deviation and (**i**) titin volume per cell quantified from fluorescent images of titin-GFP reporter (n ≥ 6). (**j**) Vinculin colocalization with titin per cell quantified from titin and vinculin images (n ≥ 6). All data presented as mean ± std; * p < 0.05.
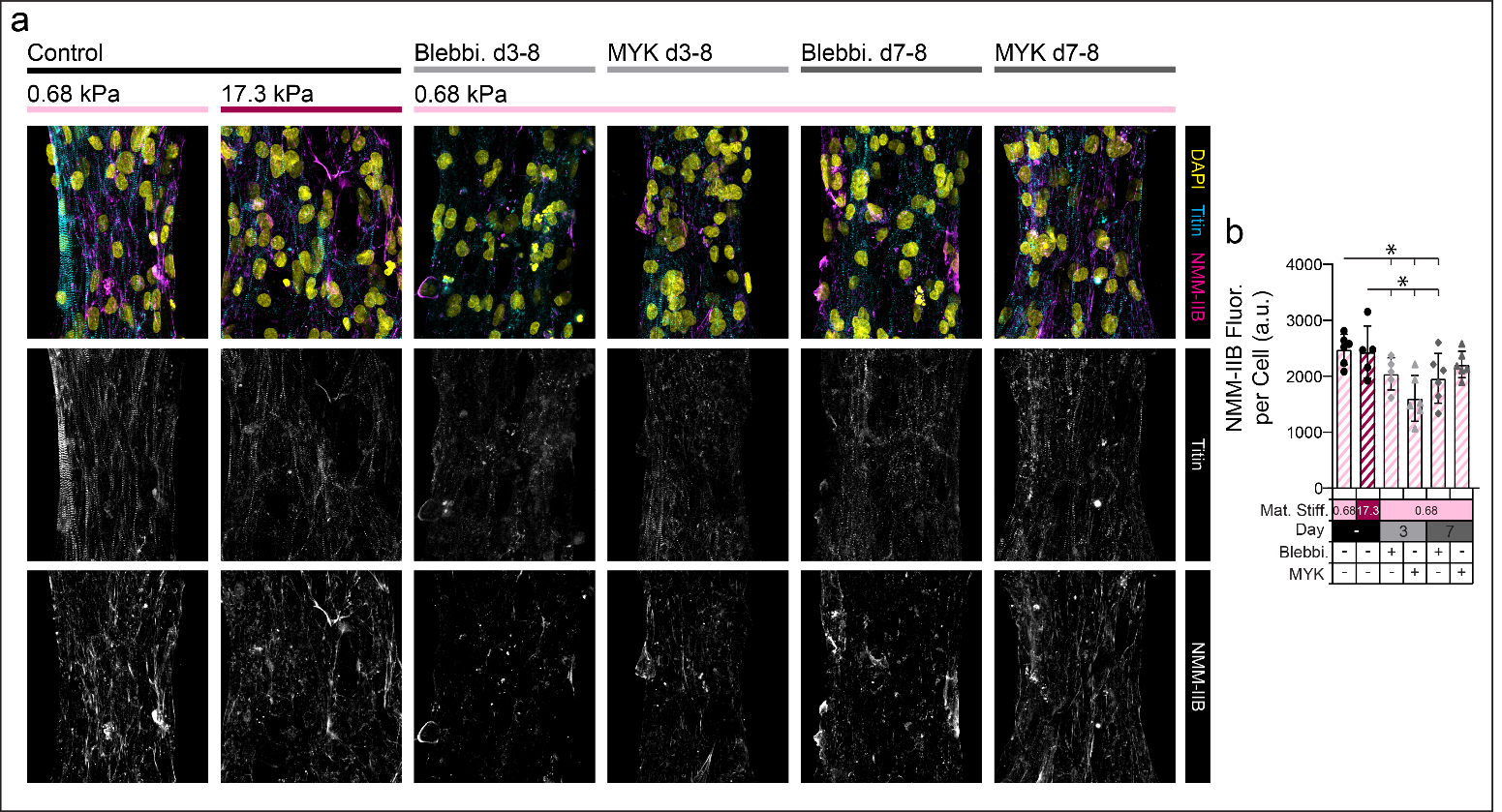


**Figure S17: Treatment with myosin inhibitors at day 3 decreases non-muscle myosin IIB (NMM-IIB) expression. (a**) Confocal fluorescent images of fibroTUG tissues treated with blebbistatin (50 µM) or mavacamten (500 nM). (**b**) Quantification of NMM-IIB expression per cell (n ≥ 6). All data presented as mean ± std; * p < 0.05.

**SUPPLEMENTAL VIDEOS**

**Supplemental Video S1:** Representative brightfield video of an array of contracting fibroTUG tissues formed on aligned, soft (0.68 kPa) fiber matrices suspended between soft (0.41 N/m) posts.

**Supplemental Video S2:** Representative brightfield videos of contracting fibroTUG tissues formed on aligned fiber matrices of varying stiffness suspended between soft (0.41 N/m) posts.

**Supplemental Video S3:** Representative brightfield videos of contracting fibroTUG tissues formed on random fiber matrices of varying stiffness suspended between soft (0.41 N/m) posts.

**Supplemental Video S4:** Representative brightfield videos of contracting fibroTUG tissues formed on aligned, soft (0.68 kPa) fiber matrices suspended between posts of varying stiffness.

**Supplemental Video S5:** Representative brightfield videos of contracting fibroTUG tissues formed on random, soft (0.68 kPa) fiber matrices suspended between posts of varying stiffness.

**Supplemental Video S6:** Representative videos of calcium fluxes in fibroTUG tissues treated with Cal520-AM dye formed on aligned fiber matrices of varying stiffness suspended between soft (0.41 N/m) posts.

**Supplemental Video S7:** Representative videos of calcium fluxes in fibroTUG tissues treated with Cal520-AM dye formed on aligned, soft (0.68 kPa) fiber matrices suspended between posts of varying stiffness.

**Supplemental Video S8:** Representative videos of calcium fluxes in fibroTUG tissues treated with Cal520-AM dye formed on random, soft (0.68 kPa) fiber matrices suspended between posts of varying stiffness.

**Supplemental Video S9:** Representative brightfield videos of contracting fibroTUG tissues formed on aligned, soft (0.68 kPa) fiber matrices suspended between soft (0.41 N/m) posts before and after isoproterenol treatment.

**Supplemental Video S10:** Videos depicting computational models of a fibroTUG tissue. The top video shows measured tissue displacements used to verify the model’s accuracy while the bottom videos show maps simulated tissue displacements and sarcomere strain.

**SUPPLEMENTAL METHODS**

**Tendon Progenitor Cell Isolation and Culture**

Tendon progenitor cells were isolated and cultured as previously described^[1]^. For all animal procedures, the Institutional Animal Care and Use Committee (IACUC) guidelines for survival surgery in rodents and the IACUC Policy on Analgesic Use in Animals Undergoing Surgery were followed (Protocol #PRO00009868). Murine cells used in this work were harvested from 12x 6-9 week-old C57BL/6 mice (6 female,6 male). A ScxGFP mouse colony was maintained by breeding ScxGFP+/− heterozygotes modified from a C57BL/6 background with WT C57BL/6J mice, and progeny were genotyped (ScxGFP+/-versus ScxGFP−/−) using a Dual Fluorescent Protein Flashlight (Model DFP-1, Nightsea, Lexington, MA). Tail tendons were removed from euthanized mice and then encapsulated in a 2 mg mL-1 type I collagen hydrogel^[2]^. Encapsulated tissues were cultured in an incubator (37°C and 5% CO2) in DMEM containing L-glutamine (ThermoFisher, Waltham, MA), 1 v/v% penicillin/streptomycin/fungizone, and 10 v/v% fetal bovine serum for 10 days to allow tendon progenitor cells (TPCs) to migrate from tendons into the collagen gel. Following isolation and expansion of TPCs, collagen gels were digested in 0.25 mg mL-1 collagenase from C. histolyticum with 0.025 w/v% trypsin-EDTA. The resulting slurry was filtered through a cell strainer and then plated. Adherent TPCs were cultured in basal media, and cells at passage 1 were used for all experiments.

**SUPPLEMENTAL REFERENCES**

[1] R. N. Kent, M. Said, M. E. Busch, E. R. Poupard, A. Tsai, J. Xia, D. L. Matera, W. Y. Wang, S. J. DePalma, H. L. Hiraki, et al., *Adv Funct Mater* **2022**, *32*, 2207556.

[2] S. K. Denduluri, B. Scott, J. D. Lamplot, L. Yin, Z. Yan, Z. Wang, J. Ye, J. Wang, Q. Wei, M. K. Mohammed, et al., *Tissue Eng Part C Methods* **2016**, *22*, 280.
